# Fear in a Handful of Dust: The Epidemiological, Environmental, and Economic Drivers of Death by PM_2.5_ Pollution

**DOI:** 10.3390/ijerph18168688

**Published:** 2021-08-17

**Authors:** James Ming Chen, Mira Zovko, Nika Šimurina, Vatroslav Zovko

**Affiliations:** 1 College of Law, Michigan State University, East Lansing, MI 48824, USA; 2 Ministry of Economy and Sustainable Development, 10000 Zagreb, Croatia; mira.zovko@mzoe.hr; 3 Faculty of Economics & Business, University of Zagreb, 10000 Zagreb, Croatia; nsimurina@efzg.hr; 4 Faculty of Teacher Education, University of Zagreb, 10000 Zagreb, Croatia; vatroslav.zovko@ufzg.hr

**Keywords:** air pollution, particulate matter, PM_2.5_, public health, environmental Kuznets curve, machine learning, supervised learning, unsupervised learning, clustering, manifold learning, dimensionality reduction, principal component analysis, European Union

## Abstract

This study evaluates numerous epidemiological, environmental, and economic factors affecting morbidity and mortality from PM_2.5_ exposure in the 27 member states of the European Union. This form of air pollution inflicts considerable social and economic damage in addition to loss of life and well-being. This study creates and deploys a comprehensive data pipeline. The first step consists of conventional linear models and supervised machine learning alternatives. Those regression methods do more than predict health outcomes in the EU-27 and relate those predictions to independent variables. Linear regression and its machine learning equivalents also inform unsupervised machine learning methods such as clustering and manifold learning. Lower-dimension manifolds of this dataset’s feature space reveal the relationship among EU-27 countries and their success (or failure) in managing PM_2.5_ morbidity and mortality. Principal component analysis informs further interpretation of variables along economic and health-based lines. A nonlinear environmental Kuznets curve may describe the fuller relationship between economic activity and premature death from PM_2.5_ exposure. The European Union should bridge the historical, cultural, and economic gaps that impair these countries’ collective response to PM_2.5_ pollution.

## 1. Introduction

### 1.1. PM_2.5_ Air Pollution

Accelerated urban development and modernization have turned air pollution into a global environmental and public health issue. According to the World Health Organization (WHO), air pollution is strongly connected to the health status of the population [1]. Air pollution has surpassed poor sanitation and a lack of drinking water as the leading environmental cause of premature death [2]. Air pollution also puts considerable pressure on household, hospital, and public budgets [3].

Air pollutants include gaseous pollutants and microscopic particulate matter (PM) that contains various toxic substances, including free radicals, metals, and organic compounds such as black carbon and mineral dust [4]. As a major component of outdoor air pollution, PM includes PM_10_ (particles ≤ 10 µm in diameter), PM_2.5_ (particles ≤ 2.5 µm in diameter), and PM_0.1_ (particles ≤ 0.1 µm in diameter).

Absent direct or indirect human activity, PM is emitted by natural sources such as volcanos and earthquakes, sea spray, wildfires, and desert dust [5]. Most PM air pollution, however, results from human activities such as combustion of fluid fossil fuels, road traffic, residential heating, construction and demolition, and industrial processes [6]. With a half-life as long as several weeks, PM can travel more than 1000 km [7]. As a result, transboundary air pollution can affect locales far from sources of PM [8].

WHO [9] and the European Union (EU) [10] have set standards for airborne pollutants and issued guidance on threshold limits. These global and European environmental policies are highly cost-effective in reducing health risks from PM. Compared to 1990, the EU’s environmental policies reduced the number of premature deaths attributed to PM_2.5_ pollution by one million [11].

The International Agency for Research on Cancer has classified air pollution in general, including PM, as a source of human carcinogens [12]. Exposure to airborne PM is closely related to the incidence of acute and chronic diseases, primarily of the cardiovascular and respiratory systems [13,14,15,16]. The Global Burden Disease Study 2017 attributes 38.44 deaths per 100,000, from all causes, to ambient PM pollution [16].

Relative to larger forms of particulate matter, PM_2.5_ has a deeper impact on mortality and morbidity [17]. Thanks to its small diameter, PM_2.5_ can bypass the filtration of nose hair, accumulate in the bronchi and lungs, and diffuse from the lungs to the bloodstream and other parts of the body. Among airborne pollutants, PM_2.5_ exhibits some of the strongest links to morbidity and premature mortality from respiratory diseases and disorders, heart diseases, and cancer [18]. In 2018, the European Environment Agency estimated that long-term exposure to PM_2.5_ accounted for approximately 379,000 premature deaths in what were then the 28 member states of the EU [6].

PM_2.5_ exerts enormous pressure on the cardiovascular and respiratory system in all population groups and contributes to morbidity, hospital admissions, and premature deaths [19,20,21,22]. Each increase of 10 μg/m^3^ in PM_2.5_ is associated with a 16 percent increase in mortality from ischemic heart disease and a 14 percent increase in mortality from stroke [23]. Long-term exposure to elevated levels of PM_2.5_ has been related to the significant incidence of chronic obstructive pulmonary disease (COPD) and asthma [24]. Extensive evidence shows positive correlation between elevated levels of airborne PM_2.5_ and the incidence of lung, tracheal, and bronchial cancer [25,26].

Even at very low levels, PM_2.5_ pollution harms health [18]. At each level of exposure, elderly people are more susceptible than the general population. Because comorbidities place the elderly at higher risk, PM_2.5_ exposure may even be fatal [27]. Increases in deaths among the elderly are strongly related to increased PM_2.5_ levels [28].

PM_2.5_ pollution profoundly harms health in the EU-27, home to almost 448 million inhabitants. In 2019, 20.3 percent of the EU-27 population was over 65 years old [29]. The high proportion of elderly in the European population heightens the prevalence of negative health conditions (such as chronic diseases) as well as the adverse health effects of pollution [27].

The leading causes of premature death in the EU-27 are circulatory diseases, cancer, and respiratory diseases, especially during winter [30]. The vast majority of respiratory diseases affect people older than 65 years. The health of elderly people is largely determined by pre-existing comorbidities. Individual behaviors also influence exposure and sensitivity to environmental stressors [31]. Poor diets, obesity, sedentary lifestyles, and smoking all contribute to disease. These behaviors are especially common among people with lower socioeconomic status [18].

Aging as a global phenomenon portends a new demographic future. The United Nations projects that the elderly population will grow from 703 million in 2019 to 1.5 billion in 2050 [32]. That year, “one in six people in the world will be aged 65 years or over” [32] (p.2). This demographic shift is expected to put pressure on governments and public expenditures, primarily through premature deaths and lost productivity. Reduced exposure to PM_2.5_ should also reduce health costs for the elderly.

Mortality in the elderly caused by PM_2.5_ also exacerbates socioeconomic burdens. WHO reports significant social inequalities in environmental exposure between and within countries [1]. Relative to their western counterparts, eastern European countries carry a higher burden of diseases and premature deaths associated with air pollution [18].

Air quality is often worse in places where socioeconomically disadvantaged people live [33,34]. People of lower socioeconomic status are likelier to use solid fuels combined with inefficient heating devices, which degrades outdoor and indoor air quality [18,35]. Residential combustion of solid fuels is a major source of many air pollutants, including PM_2.5_ [11]. Drivers of environmental inequality can expose the elderly in the poorer countries of the EU-27 to greater environmental and social deprivation.

PM_2.5_ emissions also drive global climate change. Some substances contributing to atmospheric warming, such as black carbon, are a component of PM_2.5_ [36,37,38]. Carbonaceous aerosols from agricultural waste and wood-fuel burning, primarily by households, are dominant PM_2.5_ emissions, which stimulate radiative forcing on a regional scale [39]. Effective containment of PM_2.5_ requires the coordination of international and local efforts.

Generally, two types of health-related costs arise from environmental pollution: market and non-market costs [40]. Market costs comprise losses in productivity due to illness (opportunity costs) and health care costs, which in turn include the administrative costs of hospital admissions and the use of medical technologies or pharmaceuticals (resource costs).

Non-market or welfare costs are related to premature death and disutility because of illness or care for others. Welfare costs include not only the direct cost of premature deaths but also the cost of morbidities [41]. Apart from welfare losses in public health, pollution degrades ecological systems that support human health, such as forests or green urban infrastructure. Air pollution may reduce productivity or even increase criminal and unethical behavior [42]. Welfare costs are usually higher than market costs [43].

### 1.2. The Environmental Kuznets Curve

This article strives to isolate the relationship between the economic and public health consequences of PM_2.5_ pollution. Over the past three decades, an extensive literature has estimated the health costs of PM_2.5_ pollution. Even apart from the intrinsic value of life and health, public health expenditures suffice as a motivation to mitigate PM_2.5_ pollution. By reducing PM_2.5_ emissions and exposure, countries can reduce the burden of stroke, heart disease, lung cancer, and chronic and acute respiratory diseases, including asthma.

One study has projected that a 1 μg/m^3^ decrease in annual mean PM_2.5_ levels would increase Europe’s gross domestic product (GDP) by 0.8 percent, roughly EUR 200 per capita per year (in 2017 terms) [44] (p. 7). Direct economic benefits from air pollution control policies greatly exceed investments in the air quality. The balance of costs and benefits becomes even more favorable once the substantial benefits of avoided premature deaths from PM_2.5_ pollution are considered.

Although economic development and air pollution appear closely linked, there is no clear consensus on the nature of that relationship. An extensive economic literature describes an environmental Kuznets curve [45,46,47,48,49,50,51,52]. The concept represents an extended analogy to Simon Kuznets’s observation that inequality tends to increase during early stages of economic development and then to decrease as the economy matures [53].

In readily visualized mathematical terms, environmental Kuznets curves fall into three broad categories [54] (pp. 487–495); [55] (pp. 3–5):

(a) An inverted U-shaped relationship represents the canonical environmental Kuznets curve. The inverted U describes environmental degradation in three stages. At first, increasing pollution from accelerated exploitation of natural resources shows a scale effect. A composition effect then takes over, emphasizing cleaner activities in production. During this intermediate stage, the pollution rate stagnates despite economic growth. A final techniques effect takes hold as increased economic development replaces obsolete technologies with cleaner ones and reduces pollution [56,57,58,59]. An alternative account for the emergence of the inverted U-shaped curve attributes superior environmental performance at higher income levels to environmental transition theory, by which better developed economies export pollution-intensive activities to less developed trade partners [60,61,62].
(b) A monotonically increasing relationship demonstrates unequivocally positive feedback between productivity and environmental impact [63] (p. 866). There is “an increasing rate of environmental degradation per unit of income as countries become richer” [64] (p. 654).
(c) An N-shaped relationship combines the dynamics of the inverted U and the monotonic increase. At lower levels of development, the inverted U describes the relationship between economic productivity and environmental degradation. Beyond a certain income level, however, development exerts monotonically increasing pressure on the environment [63] (p. 865); [65,66,67].

**Figure 1:**
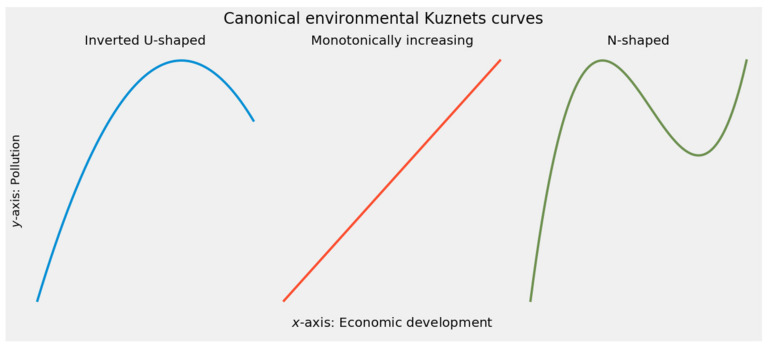
Canonical environmental Kuznets curves.

Indeed, the three canonical shapes can emerge through polynomial regression of a degree no higher than three [55] (pp. 3–5); [68] (pp. 680–682). Quadratic regression can reveal the canonical inverted U. Linear regression as a special, simplified case of polynomial regression can model a monotonically increasing relationship between development and performance. Finally, an N-shaped curve is the visual signature of cubic regression. Figure 1 depicts the three canonical shapes of the environmental Kuznets curve.

Environmental Kuznets curves necessarily simplify analysis. Each curve depicts a unidimensional relationship between economic development and a single quantity representing environmental performance. The reduction of a complex dataset with dozens of variables into a single curve trades nuance for clarity in interpretation. Indeed, proponents of regression methods designed to detect “kinks” caution against assigning undue weight to polynomial regression [51] (pp. 5–7). Other techniques, such as threshold cointegration, can also detect nonlinear relationships [69].

Any effort to construct an environmental Kuznets curve should also heed subtleties that elude this sort of analytical simplification. The shape of the curve and (consequently) the nature of the underlying relationship between economic and environmental factors vary dramatically by country [70], especially when a survey covers Africa [71] or some other slice of the developing world [72]. Divergent outcomes should also be expected within the European Union, whose member states span a wide range of incomes and developmental trajectories [73,74].

Much of the diversity in environmental Kuznets curves arises from demographic differences [75]. Even within the same country, curves can differ by pollutants, as in the case of CO_2_ versus SO_2_ and PM_10_ in Turkey [63] (pp. 865–866) or SO_2_ versus wastewater in China [76]. Perhaps most intriguing of all, and reminiscent of the field’s origins in Simon Kuznets’s work, is the suggestion that countries have some ability to exchange environmental degradation for economic equality, or economic inequality for superior environmental outcomes [72]. Optimizing environmental quality and economic equality simultaneously does not appear feasible.

Finally, any suggestion that environmental Kuznets curves must be confined to plotting raw levels of pollution against income, wealth, or other economic indicators is belied by the literature. Many authors directly plot life expectancy, healthy life expectancy, avoided mortality and morbidity, and other explicit measures of human welfare as dependent variables in environmental Kuznets curves [68,77,78]. It is now possible to speak of a “health Kuznets curve” [79,80]. This article follows the environmental branch of this emerging tradition, without fully crossing over into health-based applications having no direct connection to pollution or other environmental factors, such as cancer [81] or obesity [82].

### 1.3. An Overview of This Article

This article evaluates interactions among epidemiological, environmental, and socioeconomic drivers of PM_2.5_ pollution in the European Union. Its data covers years from 2008 to 2018. All data is collected at the level of EU-27 member states. To evaluate the impact of PM_2.5_ pollution and identify country-specific differences, this article devises an analytical pipeline that integrates conventional methods with machine learning.

This article applies both supervised and unsupervised machine learning. Supervised machine learning works alongside conventional linear models to predict PM_2.5_ mortality rates and to make causal inferences. These are the usual analytical goals for panel data. Supervised learning usually improves predictive accuracy. Machine learning’s “feature importances” complement beta coefficients and *p*-values generated by linear models.

Unsupervised machine learning provides insights along geographic lines and the conceptual boundary between economics and human health. Clustering identifies different groups among EU-27 countries according to their epidemiological, environmental, and socioeconomic traits. Decomposition and manifold learning then validate the predictions made by supervised methods and their linear counterparts. The generation and aggregation of manifolds can provide further predictions. One manifold method, principal component analysis, distinguishes the economic and health-based bases of PM_2.5_ mortality. The resulting environmental Kuznets curve simplifies and summarizes the entire study.

Part 2 of this article presents data and methods. Part 3 reports results, first for predictive linear and machine learning models, and then for unsupervised machine learning. Part 4 discusses and prescribes societal measures to ameliorate PM_2.5_ pollution. This article seeks to guide public health and environmental policy throughout Europe.

## 2. Materials and Methods

### 2.1. Data

#### 2.1.1. Dependent and Independent Variables

This article’s dataset targets PM_2.5_ mortality rates in the 27 member states of the European Union (EU-27) from 2008 to 2018 inclusive. This target variable is named *pm25_death*. Each observation covers a single year in that span for each of the countries. The dataset therefore contains 297 observations.

The dataset contains 23 independent variables:

1. *expectancy*—Life expectancy at birth (in years)
2. *poverty_threshold*—The threshold at which a single person is at risk of poverty (in euros)
3. *poverty_excluded*—The rate of risk from poverty *before* social transfers (with pensions *excluded* from the definition of social transfers)
4. *poverty_included*—The rate of risk from poverty *before* social transfers (with pensions *included* in the definition of social transfers)
5. *emissions*—PM_2.5_ emissions (in kilograms per capita)
6. *exposure*—Mean annual exposure to PM_2.5_ pollution (in µg/m^3^)
7. Five morbidity indicators—The incidence in persons older than 65 years of the following diseases:
  - *cardio_incidence*—cardiovascular disease
  - *ischemic_incidence*—ischemic heart disease
  - *copd_incidence*—chronic obstructive pulmonary disease (COPD)
  - *asthma_incidence*—asthma
  - *tracheal_incidence*—tracheal, bronchial, and lung cancer (hereinafter designated as “tracheal cancer” as shorthand covering all three types of cancer)
8. Five mortality indicators designated as *cardio_death*, *ischemic_death*, *copd_death*, *asthma_death*, and *tracheal_incidence*: The rate of premature death among persons older than 65 from each of the preceding five diseases
9. *real_gdp_pc*—Real gross domestic product (GDP) per capita
10. *health_expenditures*—Health-related government expenditures per capita
11. *environmental_taxes*—Environmentally related taxes as a percentage of GDP
12. *social_contributions*—Social security contributions as a percentage of GDP
13. *spending*—Overall government spending per capita
14. *corruption*—Corruption perception index
15. *gini*—The Gini coefficient of economic inequality

Appendix A provides further details on all of these variables and their sources.

#### 2.1.2. Endogeneity and the IVS2LS Model

We also initially gathered two data on the welfare cost of premature deaths per capita among elderly persons from PM_2.5_ and PM_10_ *combined* (*welfare_25_10*), and the welfare cost of premature deaths per capita among elderly persons from PM_2.5_ *alone* (*welfare_25*). Sources and descriptive details for these variables are also included in Appendix A. Although the White test found no statistically significant evidence of heteroskedasticity (the *p*-value for the Lagrange multiplier greatly exceeded 0.1), high Pearson’s correlation between *welfare_25* and *pm25_death* (0.940138) raised a reasonable question of endogeneity.

We built a two-stage least squares model (IV2SLS) with *exposure* as the instrumental variable for *welfare_25*, since correlation between those variables is 0.769063. Results for the Durbin (6.1673) and Wu-Hausman (110.7576) tests rejected the null hypothesis of exogeneity at every conventional confidence level. *Exposure* turns out to be a relevant and strong instrumental variable for *welfare_25*.

Consequently, we omitted *welfare_25* from this study except to the extent that it is included as the endogenous variable in the IV2SLS model. We include parameters and fitted values from the IV2SLS model in stacking generalization, the method that bridges linear regression, supervised machine learning, and unsupervised machine learning. We also omitted *welfare_25_10* in order to quell doubts over the inclusion of *any* direct measurement of welfare losses in study of mortality from PM_2.5_ pollution.

As a positive consequence of omitting both welfare variables, we were able to achieve a clean dichotomy between predominantly economic and predominantly health-related variables in constructing environmental Kuznets curves. The three poverty* variables, *emissions*, *real_gdp_pc*, *health_expenditures*, *environmental_taxes*, *social_contributions*, *spending*, *corruption,* and *gini* are treated as economic variables. *Expectancy*, *exposure*, all five morbidity variables, and all five mortality variables are defined as health-related.

#### 2.1.3. The Imputation of Missing Values

Missing values were imputed through a method combining locally weighted regression (often called LOESS or LOWESS in the statistical literature) [83,84] and polynomial splines [85,86]. In concert, these methods generate a LOESS smoothing curve approximating all available observations for each independent variable. Interpolation and extrapolation from that LOESS curve according to a polynomial spline of the appropriate order imputed all missing values. Appendix B visualizes this imputation process.

The Python computer language implements LOESS in Statsmodels and one-dimensional interpolation through polynomial splines in Scipy [87,88,89]. Despite its name, interpolation in Scipy can extrapolate values beyond either end of an incomplete series.

For each variable with missing observations, the authors used the highest-order polynomial spline that generated credible imputations. Only three variables lacked values requiring imputation. Of these, PM_2.5_ exposure was interpolated in linear fashion. Mortality from COPD and tracheal cancer was interpolated according to cubic splines. Fifty-four values for *exposure*, two for *copd_death*, and three for *tracheal_death* were imputed. None of the observations for the target variable of premature PM_2.5_ mortality was imputed.

### 2.2. Data Preprocessing and Other Preparatory Details

The gathering of data represented merely the first step. This article followed a standard pipeline for the application of supervised machine learning to panel data [90]. Although unsupervised machine learning typically does not require all of these preprocessing steps, this article uses certain outputs from supervised learning as further preprocessing so that unsupervised learning can perform predictive as well as descriptive tasks.

#### 2.2.1. Splitting and Scaling

Data preparation anticipated the application of supervised machine learning models alongside more conventional linear models. Best practices for supervised machine learning prescribe the splitting of data into randomized subsets for training and testing. Isolating data during training ensures that machine learning predictions do not merely recite assigned labels or values [91] (pp. 17–18). Reserving 25 percent of all data for testing—an arbitrary but convenient ratio—helps ensure the generalizability of supervised machine learning to data not seen during training.

Supervised machine learning achieves greater accuracy on scaled data [91] (pp. 134–142). Standard scaling reports all results in terms of Gaussian *z*-scores, or multiples of each variable’s standard deviation from its mean. Critically, the scale was drawn exclusively from training data. The scale of training data was subsequently applied to test data. The rigorous separation of data during scaling prevents inferences from the test set from leaking into training and contaminating subsequent predictions [91] (pp. 138–140).

Many applications of linear regression dispense with the splitting of data as well as standard scaling. Readers accustomed to conventional models may appreciate more detailed descriptions of the relevant data transformations. After data collection and imputation, this study began with 297 observations covering 23 independent variables and a single dependent variable. The 75/25 split in supervised learning yielded a 223 × 23 array for training and a 74 × 23 array for testing. Two scaled vectors, one with 223 values and the other with 74, expressed the ground truth of premature mortality from PM_2.5_ pollution.

This study derived all linear model parameters from the training set alone. Applying those coefficients to the holdout test set generated a smaller subset of 74 predictions. All supervised machine learning models observed the same protocol. All models reported accuracy—measured by root mean squared error (RMSE), the coefficient of determination (*r*^2^), mean bias error (MBE) [92] and [93] (p. 88), and Wilmott’s index of agreement (WIoA) [94]—separately for training and for testing.

Unsupervised machine learning involves a simpler preprocessing pipeline. The absence of target variable values—the “labels” attached to data during supervised learning—eliminates concerns over data leakage from unsupervised learning. Since all unsupervised algorithms are applied to the entire array of independent variables, splitting the data into subsets for training and testing is unnecessary. Data for unsupervised learning does undergo scaling, however, so that each dimension is expressed as Gaussian *z*-scores.

Unsupervised learning thus proceeded with a scaled but unsplit 297 × 23 array covering all values for each of the independent variables. The corresponding scaled vector of 297 observations of PM_2.5_ mortality rates remained available as the yardstick of ground truth for predictive applications of unsupervised learning. Environmental Kuznets curve analysis also used the unsplit array of independent variables and the complete ground truth vector of 297 observations.

#### 2.2.2. Beta Coefficients

The Gaussian scaling of data has one notable consequence for linear models. Scaling renders regression results from linear models in terms of standardized or beta coefficients [95]. Although some scholars discourage the use of beta coefficients in causal inference [96,97,98], this article’s linear models operate in concert with machine learning methods. Both supervised and unsupervised machine learning provide a natural check against drawing improper inferences from standardized linear models.

Moreover, the inherently dimensionless nature of beta coefficients enables direct comparisons among independent variables. For instance, beta coefficients naturally harmonize two superficially similar predictors, emissions and exposure, even though the former variable is expressed in kilograms per capita and the latter is expressed as a ratio (µg/m^3^) whose numerator is nine orders of magnitude smaller. Units as arbitrary as the designation of economic quantities in euros or U.S. dollars ultimately matter less than mathematically cogent measures of each distribution’s central tendency and variability.

#### 2.2.3. The Bias-Variance Tradeoff and Hyperparameter Tuning

Reliance on supervised machine learning demands awareness of the bias-variance tradeoff. The tension between these distinct sources of prediction error arises from an intrinsic and irreconcilable tension within machine learning. Greater inaccuracy, or bias, in the estimates of model parameters can reduce the variance among parameter estimates across samples [99]. Striking the admittedly elusive balance between bias and variance ensuring that supervised machine learning can be generalized beyond training data [100].

Bias refers to a model’s overall accuracy, particularly in training. Excessive bias results in a model that underfits its data. Highly accurate, low-bias models do not provide reliable results unless they also accommodate new, unseen data [101,102]. High-variance models tend to overfit training data. Variance therefore affects the generalizability and consistency of results. An optimally complex model strikes the ideal balance between underfitting and overfitting and minimizes total prediction error [103] (p. 107).

Bias-variance optimization in practice relies on hyperparameter tuning. Hyperparameters configure each supervised learning model according to the shape of the data or the rate at which the model during training adapts to predictive errors.

At 297 observations, the PM_2.5_ dataset is quite modest in size. The even smaller size of the 25 percent of the dataset reserved for testing (no more than 74 observations) raises the premium on cross-validation in hyperparameter optimization [91] (pp. 267–282). This technique overcomes small dataset size by using multiple “folds” of the training data as synthetic validation sets. Cross-validation facilitates experimentation with different hyperparameter settings without leaking holdout test data during training.

This dataset’s modest size did make it feasible to conduct comprehensive grid searches through each combination of hyperparameter values [91] (pp. 258–267). To ensure reproducible results, the Python script for this article set a consistent seed of 1 in SciKit-Learn’s pseudo-random number generator [104].

The success of hyperparameter testing in preventing overfitting, within individual models and overall, allays concerns over the adequacy of this dataset. Supervised machine learning results for this dataset, as reported in part 3, reveal almost no overfitting. Consistently accurate test results using holdout data vindicate reliance on this dataset. The depth of data needed for effective machine learning may be shallower than conventionally supposed. Even in extremely high-leverage applications such as the exploration of chemical space for molecules with pharmaceutical potential, “robust models can be learned from far fewer examples than has been widely assumed” [105].

Unsupervised machine learning provides further assurances that causal inferences drawn from this dataset are reliable. The harmonization of beta coefficients and *p*-values from linear models with feature importances reported by supervised machine learning models gives rise to predictive manifolds. This method generates an additional set of predictions through an ensemble of linear decomposition and nonlinear manifold learning methods. Since those predictions rely predominantly on feature weights derived from supervised machine learning, their accuracy (or its absence) sheds further light on the validity and reliability of this article’s consciously predictive models.

### 2.3. Predictive and Supervised Methods

This article deploys conventional linear models as well as supervised machine learning models for regression.

#### 2.3.1. Conventional Linear Models

This study’s baseline model is pooled ordinary least squares (pooled OLS):

(1) $$y_{it}=\beta_{0}+x_{it}^{\prime }\beta +\varepsilon_{it}$$

where $x_{it}^{\prime }$ is the array of independent variables, β is the vector of coefficients, and $\varepsilon_{it}$ is the error term [106] (p. 702) and [107] (p. 386).

Inasmuch as this study’s dataset consists of 11 yearly observations for 27 countries, fixed effects models ameliorate the possibility that the pooled OLS model might fail to detect country-specific or (perhaps less plausibly) year-specific differences [108,109]. To counter this potential source of omitted variable bias [110,111], this study deployed entity-, time-, and entity-and-time-based variants of a fixed effects model. These models will be called the FEE, FTE, and FETE models in the balance of this article.

Without loss of generality, the FEE model may be written as:

(2) $$y_{it}=\alpha_{i}+x_{it}^{\prime }\beta +\varepsilon_{it},\;\varepsilon_{it}\sim IID\left(0,\;\sigma_{\varepsilon }^{2}\right)$$

where $\alpha_{i}$ represents entity-specific heterogeneity and the expression $\varepsilon_{it}\sim IID\left(0,\;\sigma_{\varepsilon }^{2}\right)$ indicates that all $x_{it}$ are independent of all $\varepsilon_{it}$ [107] (pp. 386–388) and [112] (pp. 484–486). In practical terms, each fixed effect unit (whether a geographic entity or a year) adds a dummy variable to the model. The FTE model may be written in the same way as Equation (2), with $\gamma_{t}$ substituting for $\alpha_{i}$.

By extension, the FETE model would include both $\alpha_{i}$ and $\gamma_{t}$:

(3) $$y_{it}=\alpha_{i}+\gamma_{t}+x_{it}^{\prime }\beta +\varepsilon_{it},\;\varepsilon_{it}\sim IID\left(0,\;\sigma_{\varepsilon }^{2}\right)$$

The random effects (RE) model assumes that all factors affecting the dependent variable, but not included in the vector of independent variables, can be expressed by a random error term. By analogy to Equation (2), the RE model may be written as:

(4) $$y_{it}=\mu +\alpha_{i}+x_{it}^{\prime }\beta +\varepsilon_{it},\;\varepsilon_{it}\sim IID\left(0,\;\sigma_{\varepsilon }^{2}\right),\;\alpha_{i}\sim IID\left(0,\;\sigma_{\varepsilon }^{2}\right)$$

where $\alpha_{i}+\varepsilon_{it}$ represents an error term containing two components, a time-invariant specific component and a remainder component, presumably uncorrelated over time. In addition to being mutually independent, each of these components is also independent of *x_it_* for all *I* and all *t* [107] (p. 347).

As applied to this dataset, the Hausman test reports *χ*^2^ = 167.517. That result’s extremely low *p*-value counsels adoption of a fixed effects model over the random effects model [113] (pp. 1251–1252). Although the Hausman test favors FEE over RE, other sources advise the use of both fixed and random effects when panel data covers a defined number of countries over a defined time period [112] (pp. 495–496). Fixed effects may avoid potential biases arising in the RE model from correlations between predictive variables and omitted attributes of each country [114] (p. 10).

A simplified specification of the instrumental variable two-stage least squares (IV2SLS) model flows from pooled OLS and Equation (1):

(5) $$y_{it}=\beta_{0}+x_{it}^{\prime }\beta +\varepsilon_{it}+y_{jt}^{*}\beta^{*}+\zeta_{jt}$$

where $y_{jt}^{*}\beta^{*}$ designates the estimation of a vector of endogenous variables (which are correlated with residuals from the error term of an OLS estimate of $y_{it}$) from a vector of instrumental variables and $\varepsilon_{it}+\zeta_{jt}$ together represent the composite error term, combining error from the underlying OLS estimation and the estimate of the instrumental variables [112] (pp. 528–529). Fitted values for the instrumental variable(s) replace the endogenous variable(s). IV2SLS works because composite error $\varepsilon_{it}+\zeta_{jt}$ has zero mean and is uncorrelated with instrumental as well as exogenous variables.

This study’s treatment of *exposure* as an instrumental variable is analogous to an intention-to-treat parameter in program evaluation literature [112] (p. 527). Exposure to PM_2.5_ serves a proxy for both premature mortality (our target variable) and societal welfare loss (an endogenous predictor), because exposure to pollution is a prerequisite for both phenomena.

Especially in policymaking settings, regression serves two distinct purposes [115]. Each corresponds to the two sides of a regression equation. Some applications emphasize the coefficients of a regression model’s explanatory variables. In other settings, decision-makers focus on the fitted value of the dependent variable [116] (p. 1445).

Even if the Hausman test recommends fixed over random effects, the RE model’s parameters and predictions are worth consulting. Like the IV2SLS model treating *exposure* as an instrumental variable for welfare losses attributed to PM_2.5_ pollution, the RE model’s fitted values may inform a stacking blender. Unlike the IV2SLS model, however, the RE model does generate coefficients and *p*-values in vectors that can be harmonized with the interpretive results of supervised machine learning models. Though the RE model may not yield consistent results in the strictest econometric sense, its parameters can be understood in stacking generalization. For these reasons, we shall retain the RE model.

None of the three fixed effects models (FEE, FTE, and FETE) or the RE model is expected to achieve superior accuracy vis-à-vis pooled OLS. *r*^2^ in IV2SLS is invariably smaller than in pooled OLS because OLS minimizes the sum of squared residuals [112] (p. 527). All alternative models, however, should be more conservative in attributing statistical significance to any predictive variable. They are likelier to give consistent estimates. Given this dataset’s high degree of dimensionality relative to the modest depth of its data, the fixed and random effects models guard against improper inference from a pooled OLS model not consciously accounting for entity- or time-specific effects.

#### 2.3.2. Supervised Machine Learning

Few if any analytical tools outperform linear models in explaining the relationship of predictive variables to fitted results. Machine learning alternatives, however, often outperform linear regression in predicting fitted values that reflect the ground truth. Even where the interpretive benefit of statistically significant coefficients outweighs the value of predictive accuracy, goodness of fit is far from irrelevant. Indeed, model accuracy plays a pivotal role in unsupervised machine learning.

This article therefore deploys machine learning models alongside linear models more commonly applied in econometrics and public health. Supervised machine learning models are robust in the presence of outliers. Indeed, machine learning heralds a systematic preference for the retention of outliers in regression [90] (pp. 13–14). Machine learning is also quite forgiving of misspecification or the inclusion of weakly predictive or non-predictive variables. Machine learning alternatives to conventional linear methods are often (though not invariably) more accurate. Even where accuracy does not improve, supervised machine learning may be justified as an exhaustive robustness check.

The classification and regression tree (CART) algorithm supports a dazzling constellation of methods [117,118]. Ensemble methods aggregate numerous decision trees [119]. Instead of searching for the best feature when splitting a node, random forests search for the best feature within a random subset [120,121]. They require the tuning of only two hyperparameters: the maximum number of features in a randomized tree and each tree’s maximum depth. Randomizing the thresholds for each feature, as opposed to searching for the optimal threshold, yields an even more stochastic algorithm called extremely random trees, or extra trees [122].

Boosting represents a special class of ensembles that combine weak learners into a strong learner [123]. Each step in sequential training corrects mistakes by preceding predictors [124] (p. 199). Hyperparameters control the learning rate as well as the depth and growth of decision trees [124] (p. 204). The canonical boosting method, AdaBoost, uses decision stumps, or decision trees truncated after a single split [125] (p. 125). After each training instance, AdaBoost updates weights for each predictor.

Among the many variants of gradient boosting [126], this article implements three methods. First, it applies SciKit-Learn’s native gradient boosting regressor. The second method, XGBoost, overcomes limits on speed and scalability that have plagued other boosting algorithms [127,128]. The final gradient boosting method, LightGBM, aggressively bundles mutually exclusive features with the largest gradients to achieve efficient and scalable gradient boosting for high-dimension, large datasets [129]. LightGBM has won favor in financial forecasting [130]. The use of LightGBM in forecasting wind power production suggests its broader applicability in environmental economics and public health [131]. Simultaneous applications of XGBoost and LightGBM, sometimes alongside other implementations of gradient boosting, have begun to emerge [132,133].

This dataset’s modest size may fall short of the scale at which boosting models work best. This is especially true of LightGBM, which typically requires a deeper set of features and observations. The no-free-lunch theorem of computer science, however, posits that it is impossible to know in advance whether a particular model will outperform another at a particular task [134]. Python’s implementation of all linear and machine learning models also makes it relatively easy to subject a single dataset, once it exits the preparatory pipeline for imputing, splitting, and scaling data, to a wide array of models.

In total, this article implements six linear models and six machine learning models:

- Linear models:
  ○ Pooled OLS
  ○ Fixed entity effects (FEE)
  ○ Fixed time effects (FTE)
  ○ Fixed entity and time effects (FETE)
  ○ Random effects (RE)
  ○ Instrumental variable/two-stage least squares (IV2SLS)
- Machine learning models
  ○ Conventional decision tree ensembles
    ▪ Random forests
    ▪ Extra trees
  ○ AdaBoost
  ○ Gradient boosting models:
    ▪ Gradient boosting in SciKit-Learn
    ▪ XGBoost
    ▪ LightGBM

#### 2.3.3. Interpreting and Synthesizing Machine Learning through Feature Importances

Critics denigrate machine learning as a heuristically opaque, uninterpretable black box [135]. Conventional statistical tools such as the scale and sign of coefficients, *p*-values, and confidence intervals are unavailable in machine learning. Supervised machine learning, however, can be interpreted on its own terms. Indeed, this study breaks new ground in harmonizing the interpretation of linear and machine learning regression models.

Ensemble and boosting models based on decision trees quantify the contribution of each predictive variable. Recall that boosting methods such as XGBoost and LightGBM are special instances of decision tree ensembles. By taking the weighted averages of training samples associated with nodes across all trees in an ensemble, feature importances report each regressor’s contribution to the model’s predictions [124] (pp. 198–199) and [136]. As with any other vector of probabilities, the sum of any model’s feature importances is 1.

This article implements a novel method for bridging the interpretive gap between linear regression and machine learning [137]. Rather than insisting that machine learning speak in terms of coefficients and arbitrary thresholds of significance, we translate those attributes of linear regression into the mathematical logic of machine learning.

Expressing a linear model in terms of beta coefficients renders each parameter according to dimensionless Gaussian *z*-scores. Taking the absolute value assigns each beta coefficient a weight corresponding to its distance from that variable’s mean. So transformed, beta coefficients attain the non-negativity demanded of any distance metric.

Moreover, the corresponding vector of *p*-scores for each independent variable can be treated in continuous rather than categorical terms. Conventional treatment of *p*-values creates a small constellation of stars indicating significance at arbitrary thresholds such as 0.01 or 0.05. Nevertheless, there is a palpable difference between *p* = 0.12 and *p* = 0.99, even if neither value falls below even the most generous significance level by social science conventions. Subtracting the vector of *p*-values from 1 provides a principled and credible basis by which to discount the absolute value of the vector of beta coefficients.

Those two vectors, *β* and *p*, can in turn be normalized so that their sum of their joint transformation is invariably 1. The resulting vector of *emulated* feature importances allows every linear model to be compared to the vector of actual feature importances for an ensemble- or boosting-based machine learning model.

The emulated feature importance of each independent variable in linear regression may be expressed as:

(6) $$f_{v}=\frac{\left|\beta_{v}\right|{\left(1-p_{v}\right)}^{\gamma }}{\sum_{j=1}^{m}\left|\beta_{j}\right|{\left(1-p_{j}\right)}^{\gamma }}$$

where *β* indicates the beta coefficient, *p* indicates its corresponding *p*-value, the subscript *v* identifies each independent variable, *j* is an indexing variable, *m* indicates the number of predictors, and *γ* is an exponent. In typical practice, *γ* ∈ {1, 2}. Setting *γ* = 2, an admittedly arbitrary choice, more sharply discounts variables with higher *p*-values, which are likelier to lack statistical significance. For each *f_v_* in a linear model’s vector of emulated feature importances, a machine learning model generates a true feature importance.

#### 2.3.4. Stacking Generalization

In concert, actual and emulated feature importances enable linear and supervised machine learning models to be interpreted in complementary fashion. Once a final predictive tool, the stacking blender, enters the picture, the combined effect of all feature importances also informs unsupervised machine learning. The vector of feature importances for all predictive models—or any subset of them—emerging from the stacking blender can be used to engineer the array of predictive variables in unsupervised learning.

The primary use of stacking generalization is to aggregate predictions from other models into a global set of meta-predictions [124] (pp. 208–211) and [138]. Stacking generalization has emerged as a potent tool for “super learning” in high-stakes applications such as motion detection [139,140]. Like all other ensembles, a stacking model gracefully accommodates weaker models. Stacking often (but not invariably) delivers predictions that are more accurate than any of the tributary models fed into the meta-model.

This meta-ensemble stacks predictions from all other models (or some subset) as “level 0” in a new predictive model. Instead of the features in the PM_2.5_ dataset, stacking treats each model in level 0 as an independent variable. A meta-learner, or blender, then enters the stacking model as level 1. The blender trains on instances in training set predictions from each of the models in level 0. The trained blender can then produce its own predictions with test set predictions from the level 0 models.

The level 1 blender can be based on any regression model, linear or otherwise. Indeed, the default blender in SciKit-Learn’s built-in stacking blender is Ridge, or ℓ_2_-regularized OLS. SciKit-Learn’s stacking module, however, requires that all level 0 models be implemented in that package. Because there is no wrapper for Statsmodels or LightGBM within SciKit-Learn, this study devised its own stacking model to accommodate output from those packages. This custom-built stacking generalization model can use a decision tree ensemble such as extra trees as its blender. Aside from superlative accuracy, extra trees generates its feature importances, with one value for each model in level 0.

The power of this method will become evident in unsupervised machine learning. Like any other machine learning regression model, a stacking model has two immediate outputs: predictions and feature importances. Although the predictions can be compared immediately with predictions from any other linear or machine learning model, the interpretation of the stacking blender’s feature importances requires additional work.

Since the blender treats each model in level 0 as an independent variable, the dimensionality of its training data, its test data, and its model is equal to the number of models stacked. Its feature importances, in the instance of a grand stacking model accommodating all of this study’s predictive models, is an 11 × 1 vector. Feature importances for the predictive models in level 0 can be shaped as a 23 × 11 array, where independent variables in the PM_2.5_ dataset constitute the rows and models in level 0 constitute the columns. The dot product of a 23 × 11 array and an 11 × 1 vector is a 23 × 1 vector, the shape of feature importances from any individual predictive model.

Vectors matching the dimensionality of a dataset are vital in advanced machine learning. Feature importances can calculate weighted Euclidean distances [141] or calibrate weighted probabilistic neural networks [142]. This study can apply the 23 × 1 vector emerging from the stacking blender as a composite set of feature importances reflecting twelve predictive models. Those composite feature importances can scale each of the columns in the 297 × 23 version of the scaled PM_2.5_ dataset during unsupervised learning.

### 2.4. Unupervised Methods

#### 2.4.1. Unsupervised Machine Learning in Overview

This article deploys two forms of unsupervised machine learning: clustering and manifold learning. Unsupervised learning is distinguished from supervised learning by the absence of labels or values. Unsupervised methods operate on the complete, unsplit 297 × 23 array of independent variables.

Clustering defines distinct cohorts among the 27 member states of the European Union. If applied to all 297 observations in the PM_2.5_ dataset rather than a country-by-country aggregation, clustering can also reveal the extent to which entity effects by country or time effects by year dominate the data. Country-specific labels drawn from the clustering of aggregated data will enhance the environmental Kuznets curve.

This article uses the term “manifold learning” to refer to the broad category of unsupervised machine learning methods for reducing dimensionality. We will not draw the technical distinction between linear decomposition methods (such as principal component analysis and factor analysis) and nonlinear methods (such as multidimensional scaling) [143] A *manifold* is a lower-dimensional representation of a high-dimensional object, akin to the familiar example of a still photograph capturing a flat image of a three-dimensional object or the four-dimensional progression of that object through space and time [124] (p. 218). All forms of manifold learning described in this article yield results that can be interpreted alongside one another. Indeed, this article will aggregate all such results in a fashion similar to the stacking generalization of predictions from supervised models.

Manifold learning can operate wholly apart from clustering. One such use involves the removal of outliers before the identification of images through deep learning [144]. The canonical application of manifold learning, however, typically complements clustering to summarize and visualize high-dimensional data. This article likewise uses two- and three-dimensional manifolds of the PM_2.5_ dataset’s 23-dimensional space to visualize and interpret clustering results. As will become evident, even more aggressive reduction of dimensionality into a single vector can serve predictive purposes. At the very least, a single vector based on the economic or health-based subset of predictive variables can generate the lone independent variable used to plot the environmental Kuznets curve.

#### 2.4.2. Clustering through Affinity Propagation

Applications of clustering abound throughout the natural and social sciences [145,146,147,148]. This article relies exclusively on affinity propagation [149,150,151]. Affinity propagation finds “exemplars,” or typical cluster members, by exchanging messages between pairs of datapoints until the algorithm converges on a reliable set of exemplars and their corresponding clusters [149]. By avoiding stochastic instantiation, affinity propagation solves the conundrum of determining the ideal number of clusters [152,153].

Affinity propagation is used in the clustering of microarray and gene expression data [154,155,156] and in sequence analysis [157]. Its versatility beyond bioinformatics [158], especially in natural language processing (NLP) [159,160,161] and computer vision [162,163], suggests that this article’s mathematically modest clustering exercise can benefit from interdisciplinary cross-pollination. Despite superficial differences, language, image, and panel data are all amenable to unsupervised learning. Clustering and manifold methods that work in NLP and computer vision are almost assuredly feasible in social science.

If anything, purely numerical datasets are more tractable. Through imputation and other data cleaning methods, panels in public health, epidemiology, and related fields can be perfectly dense. Each feature has exactly the same number of observations. By contrast, the vectorization of documents generates sparse matrices that do not readily reveal semantic content. Discretionary decisions over stop words and *n*-grams are peculiar to NLP.

Similarly, panel datasets are usually less computationally intensive than high-resolution images. Though perfectly dense, image arrays are functionally, if not mathematically, sparse. Computationally meaningful portions of an image (such as eyes, nostrils, and lips in facial recognition) may be separated by vast amounts of indeterminate noise.

Panel data analysis assuredly places different weight on different features. Some independent variables inevitably contribute more than others to a model’s predictions. This study seizes upon that attribute of the PM_2.5_ dataset. Panel data analysis can exploit the scaling or outright removal of features in ways that NLP and computer vision cannot.

#### 2.4.3. Dimensionality Reduction through Manifold Learning

This article uses six methods for reducing dimensionality. Four methods—multidimensional scaling, *t*-distributed stochastic neighbor embedding, isomap, and locally linear embedding—are nonlinear methods typically regarded as manifold learning in the narrowly technical sense. Two other methods, principal component analysis and factor analysis, use linear decomposition. Those differences prove immaterial. Befitting these methods’ ultimate unity of purpose and similarity in outcomes, interlocking mathematical connections between methods efface differences between linear and nonlinear algorithms.

Perhaps the most popular tool for dimensionality reduction, principal component analysis (PCA) relies upon the singular value decomposition of data [164,165,166,167]. This study relies on SciKit-Learn’s implementation of probabilistic PCA [168,169]. Each successive dimension generated by PCA expresses progressively less variance. This property makes PCA ideal for extracting a one-dimensional vector to be projected along one axis of an environmental Kuznets curve.

Multidimensional scaling (MDS) preserves distances among observations, even as it reduces complex objects into an arbitrarily lower number of dimensions [170,171]. Because MDS produces especially vivid visual results, this manifold learning method serves as this study’s primary tool for extracting exactly three dimensions from the PM_2.5_ dataset.

*t*-distributed stochastic neighbor embedding (*t*-SNE) reduces distances between similar instances while maintaining distances between dissimilar instances [172,173,174]. Isomap, or isometric feature mapping, generates a lower-dimensional embedding that preserves geodesic distances among all datapoints [175,176,177]. It is regarded as an extension of MDS and kernel principal component analysis [178].

Locally linear embedding (LLE) preserves distances within local neighborhoods in the data [179,180]. Conceptually, LLE acts as a series of local exercises in PCA, which are ultimately evaluated on a global and nonlinear basis as the algorithm seeks the optimal embedding. At a high level of abstraction, LLE is to PCA as LOESS is to OLS regression.

Finally, factor analysis also relies upon singular value decomposition [181,182]. Factor analysis assumes that all observations are based upon some linear transformation of latent factors at a lower dimension, with added Gaussian noise. The assumption of a *standard* normal distribution, with zero mean and unit covariance, characterizes factor analysis. The further restrictive assumption of isotropic Gaussian noise, so that all diagonal entries in the dataset’s covariance matrix are the same, yields probabilistic PCA [168,169]. Factor analysis may therefore be regarded as a generalization of probabilistic PCA.

#### 2.4.4. Novel Contributions to Unsupervised Machine Learning

This study makes two innovations in unsupervised machine learning. The first involves its treatment of PM_2.5_ dataset. In addition to applying clustering and manifold learning to the unsplit 297 × 23 array of independent variables, not transformed beyond the original scaling of values for each feature, this study applies these same methods to a consciously rescaled version of the 297 × 23 array. Each of the values in the 23 × 1 vector of feature importances generated by the stacking blender corresponds to a feature in the naïvely and equally weighted, untransformed 297 × 23 array.

This scaling exercise generates a transformed 297 × 23 array susceptible to all forms of unsupervised machine learning applied to the naïve, equally weighted array. Those scalars are drawn from a vector of probabilities based on as many as 11 predictive models’ contribution to stacking generalization. An analogy to Bayesian probability may help. The naïve, untransformed array may be described as a “prior” arrangement of regressors. If so, the transformed array represents a “posterior,” accuracy-weighted array.

The separate treatment of the prior and posterior arrays leads to a second innovation in unsupervised learning. Clustering and manifold learning generate dramatically different outcomes for the prior and posterior arrays. Indeed, the appearance of almost perfectly flat regression hyperplanes in lower-dimensional manifolds derived from the posterior array invites the extension of manifold learning to its logical extreme: reduction of the dataset’s 23 dimensions to exactly one.

With one final transformation, the lone vector resulting from each exercise in manifold learning can be compared to the corresponding vector of observed PM_2.5_ mortality rates. Manifold learning based on dimensionless arrays that are scaled according to the predictions of a stacking blender may lack any connection to human-designated units. Nevertheless, the vectors produced by isomap or LLE, for instance, can be standardized so that they speak in the same Gaussian language of *z*-scores used to render this study’s ground truth.

This process may be regarded as a predictive application of unsupervised machine learning. Each predictive manifold generates a vector of *z*-scores corresponding to the 297 observations for PM_2.5_ mortality. An extension of stacking generalization yields a predictive meta-model. OLS regression of predictive manifolds produces a final, composite prediction uniting all forms of dimensionality reduction.

## 3. Results

### 3.1. Linear Models

We begin by reporting traditional parameters for linear models as guides to causal inference. In anticipation of reporting these models’ fitted values and harmonizing them with supervised machine learning models, however, we emphasize the distinction between training and test data. The baseline pooled OLS model, for instance, reported very accurate predictions on both sides of the training/test split. While *r*^2^ in training was 0.971629, the more meaningful *r*^2^ for the test set was 0.966741.

Linear models are less likely to give rise to discrepancies between accuracy statistics for training and test datasets. In machine learning, however, accuracy statistics such as *r*^2^ and RMSE must be based on holdout test data. Training set accuracy serves mostly to warn against possible overfitting and cannot be trusted as a gauge of predictive power with unseen data. Accordingly, this article will report parameters, statistical significance, and feature importances according to training data, but accuracy according to test data.

#### 3.1.1. Parameters and Statistical Significance

Table 1 reports parameters and statistical significance levels for all linear models:

According to the linear models, the most influential predictors appear to be *expectancy*, *exposure*, and *ischemic_death*. Cardiovascular morbidity and mortality achieve statistical significance in half the models. Intriguing, *cardio_incidence* and *cardio_death* have opposite signs in five of six models, with incidence negatively related to PM_2.5_ mortality.

Among economic variables, *social_contributions* registered a mild surprise. That predictor is the only other variable besides *expectancy* and *exposure* to achieve statistical significance at some conventional level in all six linear models. The *emissions* variable, classified as an economic rather than a health-based variable, is strikingly less influential than *exposure*. Only in IV2SLS is *emissions* significant, and its coefficient is dwarfed by the parameter associated with *exposure* as an instrumental variable for welfare loss.

#### 3.1.2. Fitted Values and Model Accuracy

We now examine the linear models’ fitted values and the degree of accuracy they attained in predicting PM_2.5_ death rates.

**Figure 2:**
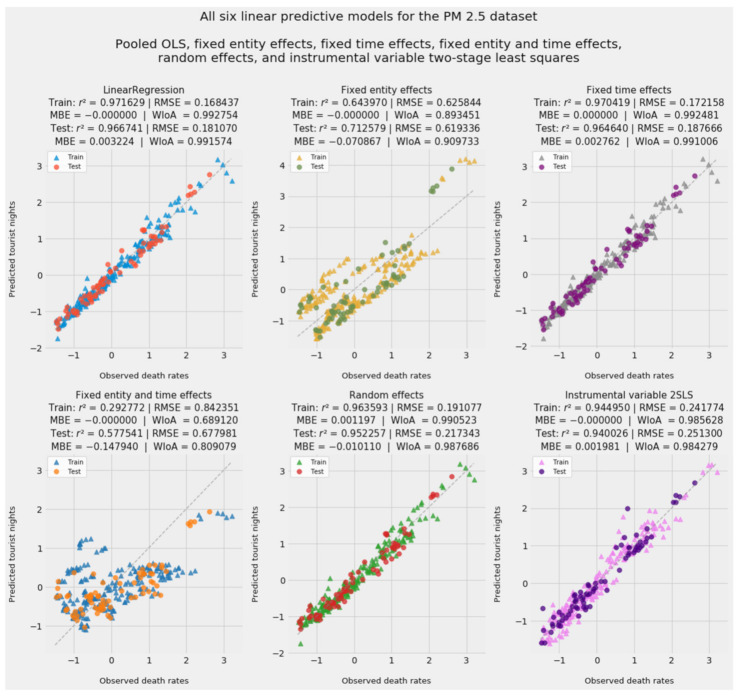
Fitted values and accuracy statistics for all linear models.

Figure 2 presents fitted values and accuracy statistics for all linear models. Ideally, *r*^2^ should approach 1 and RMSE should approach 0 as accuracy improves. Willmott’s index of agreement and MBE follow a similar distinction: an index of 1 indicates complete agreement, while departures from 0 (in either direction) indicate greater mean bias error. Negative values are expected in a model using standard-scaled data and beta coefficients; they indicate observed and fitted values below the mean.

As expected, none of the advanced econometric models outperformed pooled OLS in predicting observed PM_2.5_ mortality. Like pooled OLS, RE and IV2SLS achieved impressive and consistent accuracy at all *z*-scores in test data, from −1 to +3.

The FTE model, which omits country effects, is very close in predictive accuracy and interpretive inferences to pooled OLS. Likewise, entity effects hold sway in the FETE model and align that model with FEE. The relative contribution of entity and time effects to the linear models suggests that latent country-specific factors omitted from the pooled OLS model outweighed latent time-specific factors. Again, this is unsurprising in light of this dataset’s relatively compressed timeframe, to say nothing of geographic, economic, and social diversity within the EU-27.

The use of fixed effects models, however, is not motivated by a desire for greater accuracy. Rather, the goal is to refine the interpretive value of the baseline pooled OLS model. In particular, the coefficients associated with each entity in the FEE and FETE models will prove valuable in interpreting unsupervised machine learning results.

### 3.2. Supervised Machine Learning Models

#### 3.2.1. Fitted Values and Model Accuracy

**Figure 3:**
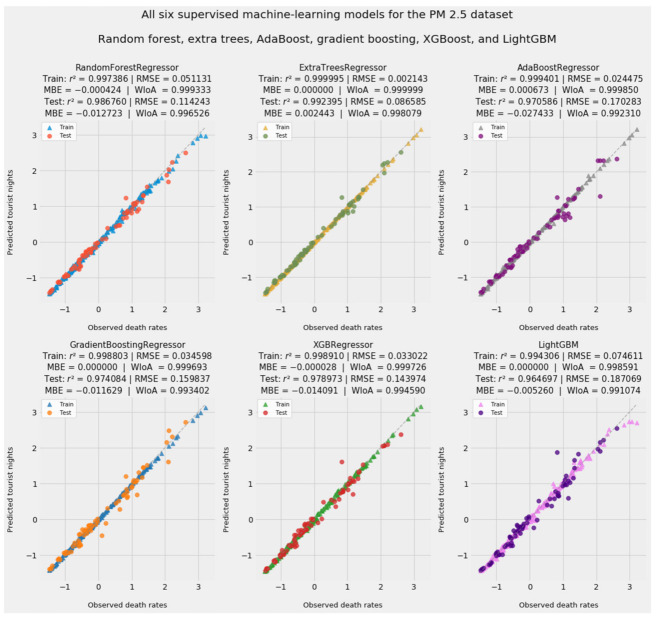
Fitted values and accuracy statistics for all supervised machine learning models.

By contrast, the raison d’être of supervised machine learning is accurate prediction. We therefore begin our review of machine learning results with fitted values and accuracy statistics, as reported in Figure 3. We then follow with a presentation of feature importances, which represent the contribution of this class of model to causal inference.

All supervised machine learning models except LightGBM outperformed the pooled OLS baseline in predicting test data. Less elaborate ensemble and boosting models rivaled or beat XGBoost and outperformed LightGBM every time. The simpler ensemble models achieve greater accuracy than XGBoost and LightGBM. The better measure of accuracy is not the marginal improvement in *r*^2^, but rather the halving of the boosting models’ RMSE. LightGBM, which consciously trades accuracy for training speed and reductions in the computational cost of calculating gradients, should not be expected to excel on smaller datasets. Despite algorithmic shortcuts of its own, XGBoost led all boosting models.

Convergence in predictive results illustrates the “unreasonable effectiveness of data” [183]. This principle of computer science recognizes that very different algorithms will attain almost identical results on complex problems—as long as there is sufficient data. Convergence in performance despite differences in algorithmic complexity suggests that data ultimately triumphs over theoretical elaboration and experimental design. It also provides further reassurance that this dataset, though small, was large enough to support causal inference through machine learning as well as linear modeling.

In addition to achieving *r*^2^ near 1 and RMSE near or below 0.1*z*, these supervised machine learning models avoid overfitting. This is an important feat. Very high test data accuracy allays concerns that this small dataset might not generalize well to unseen, holdout data. Barring sweeping, structural changes in the relationship between the predictors and PM_2.5_ mortality, supervised machine learning models trained on this dataset should perform well on economic, environmental, and epidemiological data yet to be collected.

#### 3.2.2. Feature Importances

Feature importances are to supervised machine learning as coefficients and *p*-values are to linear models. Table 2 reports the vector of feature importances associated with each of the six predictive machine learning models.

The sum of each row indicates the collective weight that a naïve ensemble of these six machine learning models would assign to a particular variable. For example, the superficially similar variables, *emissions* and *exposure*, carry dramatically different weights. AdaBoost assigns *exposure* more than a thousand times the weight it assigns *emissions*. Among the strictly epidemiological indicators of morbidity and mortality, *cardio_death* and *ischemic_death* dominate.

Since each column expresses one model’s feature importances as a vector of probabilities whose sum is 1, the distribution of weights within each column shows that model’s propensity toward either extreme of equally weighted features or a model emphasizing a single feature. The Gini coefficient and Simpson’s index of diversity [184] indicate inequality and concentration, respectively, with values of 1 indicating the assignment of all feature importance to a single variable. Simpson’s index (also known as the Herfindahl–Hirschman index in the economics of industrial organization [185,186]), $\lambda =\sum_{i=1}^{N}p_{i}^{2},\;\lambda \in \left[\frac{1}{N},\;1\right]$, has an additional helpful property: its reciprocal expresses the concentration that would be associated with a model containing $\frac{1}{\lambda }$ variables.

Table 3 reports these measures of diversity and concentration with respect to the six machine learning models.

The higher a model’s Gini coefficient or Simpson’s index, the more heavily it emphasizes features it deems important. Despite the similarity of their predictions, machine learning models vary considerably in the way they distribute feature importances. Though SciKit-Learn’s native gradient boosting regressor and LightGBM both rely in principle on gradient boosting, the SciKit-Learn package assigns nearly three-quarters of its feature importances to a single feature, *exposure*. Such concentration is suggestive of a model with no more than two predictors. LightGBM, by contrast, distributes feature importance weights so evenly that it is analogous to a model with 16 independent variables.

### 3.3. Emulated Feature Importances of Linear Models

A transformation of these beta coefficients and their corresponding *p*-values according to this article’s implementation of [137] supplements the conventional interpretation of a linear regression model. More importantly, such a transformation enables linear regression coefficients to be interpreted alongside feature importances from supervised machine learning and to be included in weighting vectors in unsupervised machine learning.

Table 4 reports emulated feature importances for all linear models.

Emulated feature importances blur the conventional distinction between linear and machine learning models. Linear models are prized for their interpretive clarity. The typical justification for machine learning assumes that CART-based ensembles and boosting models can improve accuracy. Harmonizing linear model parameters with machine learning feature importances enables supervised machine learning to contribute to interpretation and causal inference than traditionally thought.

Table 2 and Table 4 reveal surprising amounts of agreement among linear and machine learning models. For example, the extra trees and pooled OLS models assign almost exactly the same amount of inferential weight to *expectancy*, *cardio_death*, and *ischemic_death*. The IV2SLS model assigns more than 40 percent of its emulated feature importances to *exposure* (as the instrument for the endogenous variable *welfare_25*). That allocation is much closer to the allocation made by machine learning models than other linear models. At a minimum, a machine learning model such as a random forest can break the interpretive stranglehold that the more conservative FEE model might exert in an exclusively linear experimental design models.

Table 5 reports the Gini coefficient and two ways to interpret Simpson’s index for the linear models’ emulated feature importances. As a group, the linear models are closer to one another and more diverse than machine learning models. They do emphasize different predictive variables, which accounts for their far wider range of predictive outcomes.

**Figure 4:**
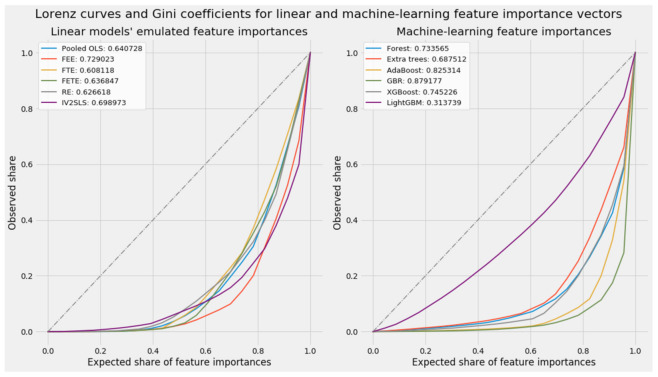
Lorenz curves and Gini coefficients for linear and machine learning models’ (emulated) feature importances. bbreviations—Pooled OLS: pooled ordinary least squares; FEE: fixed entity effects; FTE: fixed time effects; FETE: fixed entity and time effects; RE: random effects: IV2SLS: instrumental value/two-stage least squares; GBR: gradient boosting regression model.

Figure 4 portrays the Lorenz curves used to calculate the Gini coefficients for all models’ actual or emulated feature importances. These two plots visualize the greater diversity among machine learning models vis-à-vis the greater similarity in the distribution of emulated feature weights for linear models.

Ultimately, actual and emulated feature importances can be interpreted on comparable if not mathematically equal footing. The absence of negative signs, considered a vice in a traditional statistical culture that prizes heuristic ease and clarity, can prove to be a hidden virtue in the algorithmic culture of machine learning [187]. Stacking generalization of predictive results and unsupervised learning in its entirety will demonstrate the interpretive and predictive power of feature importances.

### 3.4. Stacking Generalization

#### 3.4.1. Aggregated Predictions

Stacking generalization is the culmination of supervised machine learning and this study’s overtly predictive phase. It performs two tasks. First, it aggregates all predictions, no matter their underlying model, into a final set of fitted values that can be more accurate than those of any of the models that inform it. Second, the very act of aggregation generates a weighting vector that can be used in conjunction with all emulated and actual feature importances to inform causal inference and unsupervised machine learning. This twin-pronged functionality makes stacking generalization the natural bridge between supervised and unsupervised learning.

We begin with aggregated predictions.

**Figure 5:**
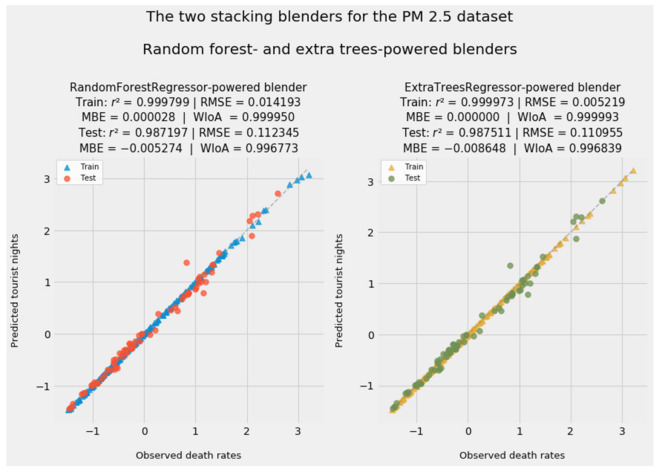
Predictions from stacking generalization, with alternative machine learning models (random forest versus extra trees) in level 1.

Figure 5 shows predictions from stacking generalization with a choice of blenders in level 1. As simple CART-based ensembles, random forests and extra trees are superbly suited to be blenders. Both blenders generated fitted values that were more accurate than predictions by 11 of the 12 models in level 0. The extra trees model finished first.

As is typical of this class of predictive models, stacking generalization achieved greater accuracy than most of the constituent predictive models in level 0. As an ensemble of ensembles, an aggregating method such as stacking or voting harnesses the Delphic wisdom of the crowd at multiple levels [124] (p. 189). The inclusion of weaker linear models such as FEE and FETE may have kept the random forest- and extra trees-based blenders from outperforming the standalone machine learning ensembles.

Stacking generalization does more than take the naïve average of level 0 predictions. Both stacking blenders, as models of models, generated their own set of feature importances. The next subsubsection addresses the significance of that feat.

#### 3.4.2. The Combined Array of Feature Importances

Aggregated predictions represent merely part of the results from stacking generalization. An aggregated vector of feature importances for emerges from the dot product of (a) the feature importances from each of the predictive models in level 0 of the stacking generalization machine and (b) the feature importances generated by the blender in level 1. This vector will prove vital as feature weights in unsupervised machine learning.

Stacking generalization begins with concatenation of feature importances reported in Table 2 and Table 4 into a grand matrix. Let **L** represent the 23 × 6 matrix of emulated feature importances for linear models, where the 23 rows designate independent variables from *exposure* to *gini* and the 6 columns designate the linear models. Let **M** represent the corresponding 23 × 6 matrix of actual feature importances for the supervised machine learning models. A grand 23 × 12 matrix **F** for all feature importances emerges from the row-wise concatenation of **L** and **M**, such that **F** = (**L** | **M**).

Let us now assign weights corresponding to each of the models corresponding to the columns in **F**. Let **W**, a 12 × 1 vector, contain those weights. The dot product, **F** · **W**, yields a 23 × 1 vector **V** corresponding to the original 23 independent variables. Vector **V** or its transpose, **V**^T^, can now be used to transform *any* matrix of the appropriate rank.

**W** could be a naïvely weighted vector of 12 consecutive values, each equal to $\frac{1}{12}$. There is a better option, however: feature importances from either of the blenders in level 1 of stacking generalization, or some weighted mean of the two.

**Figure 6:**
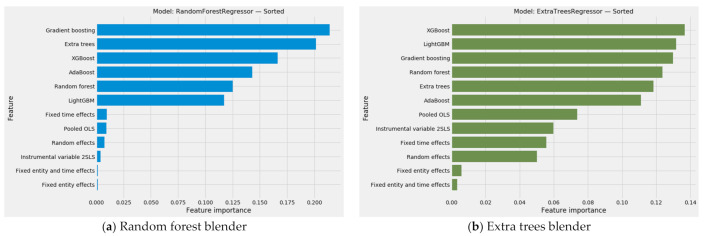
Feature importances within two stacking blenders: (**a**) random forests; (**b**) extra trees.

Figure 6 shows feature importances from the random forest- and extra-trees-based stacking blender. Although differences in accuracy provide no clear reason to prefer the extra trees blender over its random forest-based counterpart, the extra trees blender does draw from a more diverse, less concentrated of results from the constituent models in level 0. The extra trees blender had a Gini coefficient of 0.308941 and a Simpson’s index of 0.109181. The reciprocal of the Simpson’s index value, 1/*λ*, implies that this blender operated with the equivalent of 9.159139 models. For the random forest blender, *G* = 0.534323 and *λ* = 0.164151 imply a much lower number of de facto constituent models: 6.091958. Indeed, that number aligns almost perfectly with the number of models—all of them based on machine learning—given meaningful weight by the random forest blender.

As a group, the machine learning models earned a higher share of the weight within the extra trees blender’s feature importances. Each of the 12 constituent models in level 0 presumably contributes roughly 0.083 to the blender’s predictions. In reality, the blender’s underlying extra trees model assigns more than 0.1 weight to each of the machine learning models. Not one linear model attains its presumptive 0.083 share. The FEE and FETE models are given very little weight. Given the lower accuracy of those models, this reduction in weight is not surprising.

Overall, the extra trees blender preferred more accurate models—but not monotonically so. The weakest of the machine learning models, LightGBM, captured the second highest share of this vector.

#### 3.4.3. Aggregated Feature Importances

**Figure 7:**
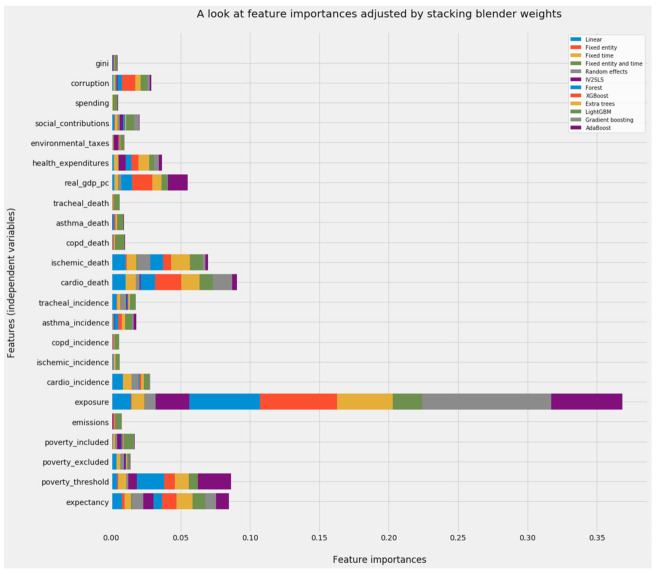
Aggregated feature importances for all predictive models and the entire PM_2.5_ dataset.

At last we take the dot product, **F** ⋅ **W**, of the combined matrix of all predictive models’ emulated and actual feature importances by the weights in the extra trees blender’s own 12 × 1 vector of importances. Figure 7 depicts **V**, the resulting vector of aggregated feature importances, which proves pivotal in unsupervised learning.

This final aggregation of feature importances confirms hints left by interpretive evidence from the constituent linear and machine learning models. *Exposure* is the dominant variable. Five other variables—*expectancy*, *poverty_threshold*, *cardio_death*, *ischemic_death*, and *real_gdp_pc*—clearly exceed each feature’s presumptive 4.35 percent share of aggregated feature importances. The disproportionate weight accorded to two economic variables (*poverty_threshold* and *real_gdp_pc*) will affect our environmental Kuznets curves.

Diversity and concentration statistics for aggregate feature importances **V** are *G* = 0.628176 and λ = 0.171340. 1/*λ* = 5.836350 implies a six-variable model, which happily corresponds to the number of features exceeding their presumptive share of weight.

The non-negativity of feature importances boasts a virtue relative to regression coefficients. This property allows feature importances, individually and collectively, to be used in scaling distances. Since unsupervised machine learning constitutes a giant exercise in computing distances across multidimensional space, this putative shortcoming in the causal inference through machine learning turns the tables.

### 3.5. Unsupervised Machine Learning on Country-Level Aggregate Data

We now turn to unsupervised machine learning. This study approaches the PM_2.5_ dataset at two levels. First, we will evaluate the mean-based aggregate for each of the European Union’s 27 member states. In very practical terms, this means working with a very small 27 × 23 array containing a single value for each country. We will then engage the data at the level of individual observations, or the full 297 × 23 scaled array.

In each instance, we will show that the application of feature importances from the stacking blender as a vector of weights will align the clusters with predictions from linear regression and supervised learning models. Indeed, realigning “naïve” clusters based on the equally weighted matrix of variables into clusters that reflect the blender-generated feature weights will generate its own set of predictions.

**Figure 8:**
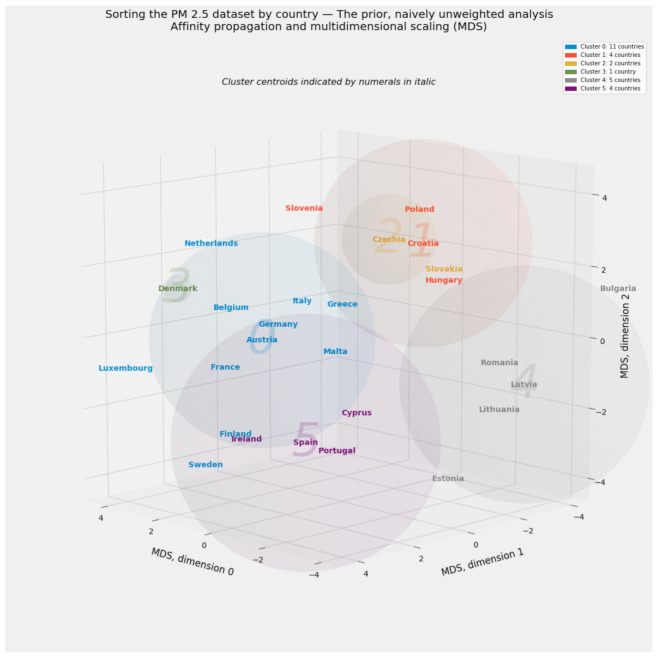
Clusters of EU countries based on naïve, unweighted country-level aggregate data.

Affinity propagation clustering according to the naïvely unweighted matrix of predictive variables yields the following clusters of countries (Figure 8):

- Cluster 0; Austria, Belgium, Finland, France, Germany, Greece, Italy, Luxembourg, Malta, the Netherlands, Sweden
- Cluster 1: Croatia, Hungary, Poland, Slovenia
- Cluster 2: Czechia, Slovakia
- Cluster 3: Denmark
- Cluster 4: Bulgaria, Estonia, Latvia, Lithuania, Romania
- Cluster 5: Cyprus, Ireland, Portugal, Spain

Each cluster’s exemplar appears at its center. Bulgaria, for example, lies a considerable distance from Latvia, which defines cluster 4. Estonia lies at the frontier between clusters 4 and 5. Slovenia likewise appears to mark the boundary between clusters 1 and 0.

The most striking impression from Figure 8’s view of EU-27 countries is the absence of any visual ordering. The qualitatively distinctive trait of these clusters is the clean separation between “eastern European” countries (defined as former members of the Warsaw Pact or the Yugoslav Federation) and their western counterparts. Cluster 2, perhaps appropriately, binds the two states born of the former federal republic of Czechoslovakia.

A more subtle distinction characterizes the western countries of clusters 0, 3, and 5. Cluster 0 is dominated by the six original signatories of the Treaty of Rome. France, Germany, Italy, and the Benelux nations comprise a bare majority of the 11 countries in that cluster. Cluster 5 includes three countries—Ireland, Portugal, and Spain—at the heart of the global financial crisis of 2008–09. All three of those countries, like Cyprus, are either islands or are bordered on the west by the Atlantic Ocean. Both those traits characterize Ireland. This geographic commonality may prove important later.

**Figure 9:**
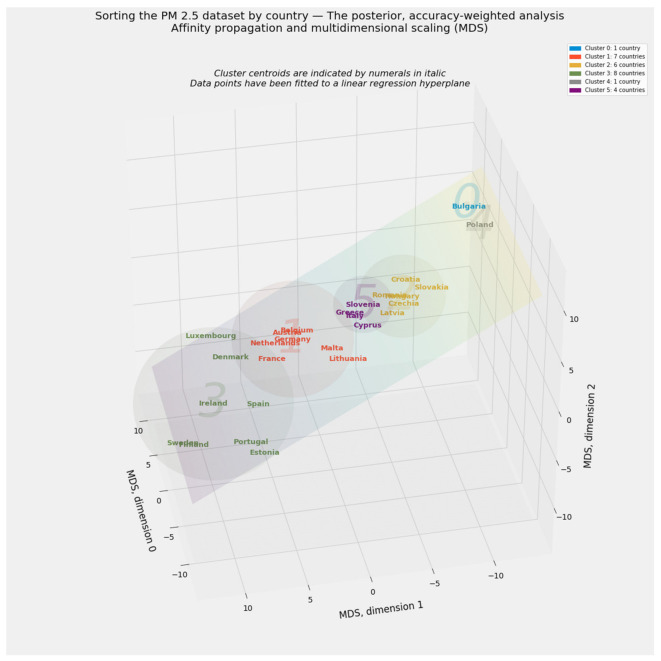
EU-27 clusters based on posterior, accuracy-weighted country-level aggregate data.

Performing the same feats of unsupervised learning—clustering through affinity propagation and manifold learning through multidimensional scaling in three dimensions—on the accuracy-adjusted array of feature importances produces a dramatically different configuration of EU-27 countries (Figure 9):

- Cluster 0: Bulgaria
- Cluster 1: Austria, Belgium, France, Germany, Lithuania, Malta, and the Netherlands
- Cluster 2: Croatia, Czechia, Hungary, Latvia, Romania, and Slovakia
- Cluster 3: Denmark, Estonia, Finland, Ireland, Luxembourg, Portugal, Spain, and Sweden
- Cluster 4: Poland
- Cluster 5: Cyprus, Greece, Italy, and Slovenia

Although new clusters 0, 2, and 4 retain an “eastern” flavor, closer examination reveals very significant departures from the strictly geographic division of the naïvely unweighted clusters. Cluster 4 from the unweighted analysis has splintered almost completely. Most strikingly, Estonia is now aligned with its Nordic neighbors, Finland and Sweden, rather than its other neighbors on the eastern shore of the Baltic Sea and south of the Gulf of Finland. Lithuania, curiously, has joined cluster 1, the new home for a quorum of the Treaty of Rome’s “Inner Six.” New cluster 2, with Croatia, Czechia, Hungary, Latvia, Romania, and Slovakia, retains a distinctively eastern flavor. Bulgaria and Poland are so far removed from every other country that they have become singletons.

Slovenia has migrated to a new cluster 5, which consists otherwise of countries we may fancifully associate with Homer’s *Odyssey*: Cyprus, Greece, and Italy. This is a striking departure from the original alignment, rivaling Estonia’s migration to new cluster 3, a low-mortality group of countries along Europe’s western and northern frontiers.

Clustering and multidimensional scaling with the posterior, accuracy-weighted array of predictive variables preserves the rough division between western and eastern Europe. The frontier between the two superclusters appears to fall between clusters 1 and 5. One of the two most marginal countries in cluster 1, Malta, would harmonize with Homeric cluster 5. Lithuania, however, would not.

The other visually striking result of clustering and manifold learning according to accuracy-adjusted weights is the rough alignment of countries according to their PM_2.5_ mortality. The clusters lie close to the flat surface of the linear regression hyperplane. Although MDS dimensions are not expressed in meaningful units, MDS dimension 1 appears to align very closely with observed PM_2.5_ mortality rates. Bulgaria and Poland dominate one extreme, while the low-mortality countries of cluster 4 lie at the opposite end. The palpably linear alignment of countries and clusters, in an analysis relying solely on a weighted array of independent variables, suggests that clustering and dimensionality reduction can serve analytically predictive as well as visually descriptive purposes.

### 3.6. Unsupervised Machine Learning on the Entire PM_2.5_ Dataset

#### 3.6.1. The Accuracy-Weighted Array Reveals Visually Linear Convergence in the Data

This subsection repeats clustering through affinity propagation and manifold learning through MDS on the PM_2.5_ dataset at the level of individual observations. Observation-level data is 11 times as deep as the country-specific aggregation. We again begin by applying affinity propagation and MDS to the naïve, equally weighted array of predictive variables. We then repeat these exercises on the posterior, accuracy-weighted array.

**Figure 10:**
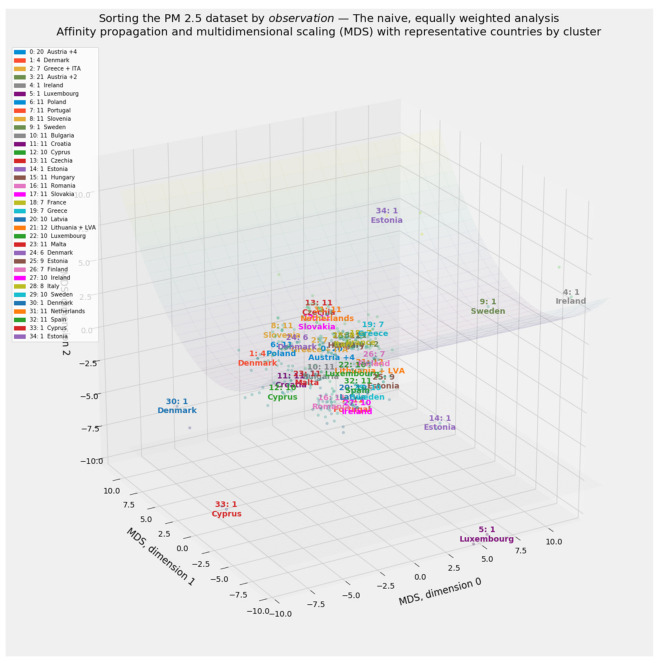
Clusters of observations within the PM_2.5_ dataset based on naïve, unweighted data.

One difference between the naïve and accuracy-weighted arrays is the number of clusters. Affinity propagation found 35 clusters within the equally weighted array and 17 clusters within the accuracy-weighted array. Figure 10 provides the naïve view.

The naïve clusters notably fail to align in a plane, let alone a line. Their most distinctive trait is the regularity with which clusters capture the exact 11 observations associated with a single country. Indeed, of the 27 EU countries, 12 formed clusters that contained only the 11 observations associated with a single country: Poland, Portugal, Slovenia, Bulgaria, Croatia, Czechia, the Netherlands, Romania, Slovakia, Malta, Hungary, and Spain. Three others—Ireland, Luxembourg, Cyprus—formed exactly one cluster of 10 observations and a second cluster consisting solely of a single observation. All 11 observations for Lithuania appeared in cluster 21, along with a single observation from Latvia. The remaining 10 observations for Latvia appeared in cluster 20. In concert, these clusters suggest that the naïvely unweighted array of predictive variables do clump together according to country-specific factors that fixed effects models are designed to expose.

**Figure 11:**
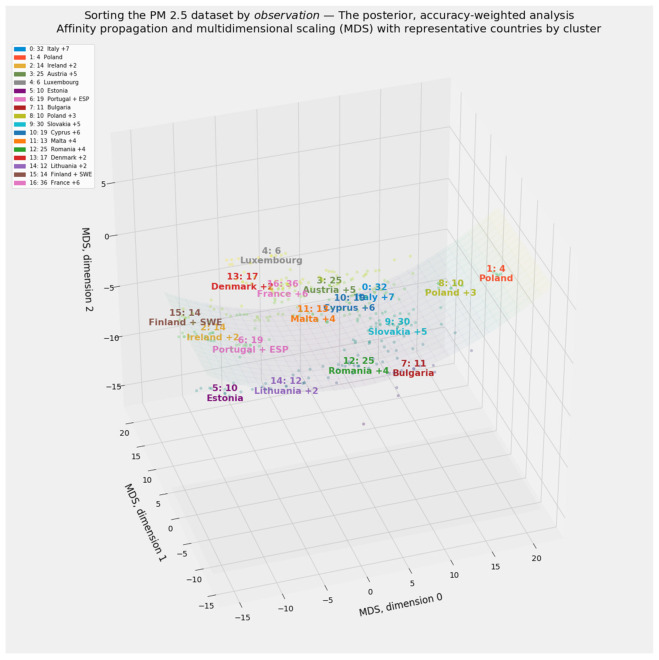
Clusters of observations within the “posterior” array of accuracy-weighted data.

Using the stacking blender’s aggregate feature importances as weights for the array of predictive variables aligns these observations and a new configuration of 17 clusters into a distinctive plane. Figure 11 illustrates this radical realignment of clusters. Even though MDS dimensions are arbitrary, most of the variance in this three-dimensional plot appears along MDS dimension 0. The quadratic regression hyperplane, though visibly curved, nevertheless remains relatively flat.

Even more gratifying is the monotonic rise of PM_2.5_ mortality rates along MDS dimension 0. PM_2.5_ mortality, after all, is this study’s target variable. The left side of this three-dimensional representation of the accuracy-weighted dataset is dominated by low-mortality countries such as Finland, Sweden, and Estonia. Romania, Slovakia, Bulgaria, and Poland dominate the right side.

The linear and monotonic alignment of EU-27 countries by PM_2.5_ mortality invites a more comprehensive and aggressive application of manifold learning. If individual observations within the accuracy-weighted array of predictive factors are reduced to a single vector of values by all manifold methods at hand and then rescaled through standardization, those vectors may produce credible predictions of PM_2.5_ mortality. The next subsubsection engages in this very exercise of predictive unsupervised learning.

#### 3.6.2. Predictive Unsupervised Learning

It is worth recalling the six linear and nonlinear methods of dimensionality reduction that Section 2.4.3 defines as “manifold learning”:

- Principal component analysis (PCA)
- Multidimensional scaling (MDS)
- Isometric feature mapping (isomap)
- Factor analysis
- *t*-distributed stochastic neighbor embedding (*t*-SNE)
- Locally linear embedding (LLE)

Figure 11 appears to display additional variance along a second dimension in the MDS manifold. This invites a seventh form of predictive unsupervised learning. By analogy to the ecliptic in astronomy, which is the plane along which the earth’s orbit travels within the solar system, MDS ecliptic accounts for two rather than merely one of the dimensions within a three-dimensional MDS projection.

“Unsupervised OLS,” or the fitted values of the linear regression of results from the foregoing seven forms of manifold learning brings the total number of unsupervised predictive models to eight. As described earlier, this methodology is analogous to stacking generalization, with the simplest form of OLS regression as the blender.

**Figure 12:**
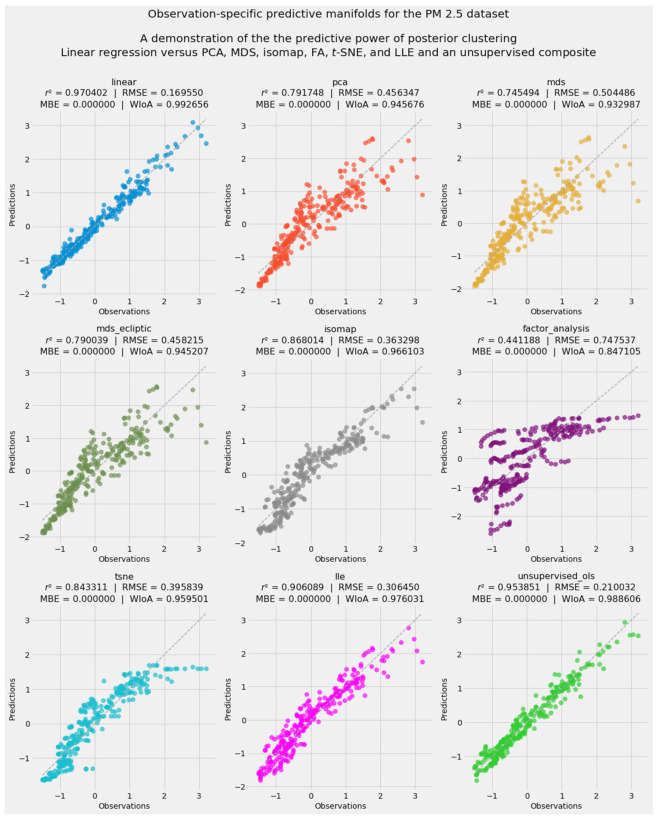
Predictive manifolds: a predictive application of unsupervised machine learning.

Figure 12 reports results from all eight of these unsupervised predictive models alongside the traditional baseline of OLS regression of the accuracy-weighted observations. The accuracy statistics accompanying each predictive manifold do not distinguish between training and test accuracy, since all forms of unsupervised learning use the full PM_2.5_ dataset without regard to a training-test split.

Like the stacking blender on which it is modeled, the aggregate “unsupervised OLS” model overcomes the inclusion of the weak factor analysis model. Unsupervised OLS ultimately registers an *r*^2^ of 0.953851 and RMSE of 0.210032. These indicators of accuracy compare favorably with the OLS regression baselines of *r*^2^ = 0.970402 and RMSE = 0.169550. The linear model has a higher index of agreement, 0.992656 to 0.988606.

**Figure 13:**
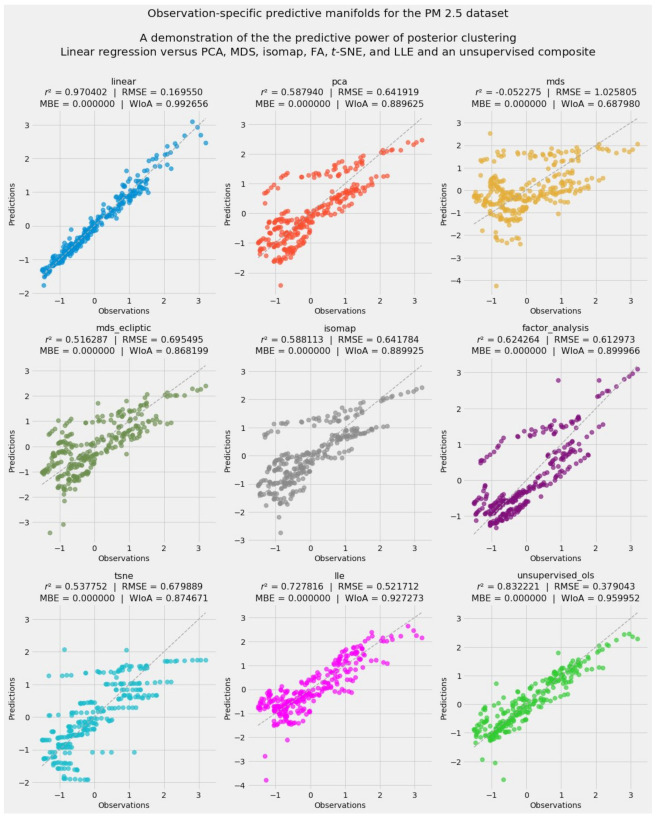
Baseline performance for predictive manifolds, based on the naïve, equally weighted array of predictive variables.

Full appreciation of improvement attained by predictive manifolds requires a look at the baseline performance that these unsupervised learning methods would have attained in the absence of any accuracy-based feature weights. Figure 13 reports the predictive manifolds generated by the naïve, equally weighted array of predictive variables (whose clusters and configuration were displayed in Figure 10).

Accuracy-based weights, in other words raised *r*^2^ by 0.122 and cut RMSE by 0.169.

In addition to producing accurate predictions, predictive manifolds further validate the idea that unsupervised machine learning can perform both of the primary functions of regression: not only prediction, but also interpretation and causal inference. Since weighting the entire 297 × 23 array of observations by aggregated feature importances achieved these predictive gains, it follows that the constituent models in level 0 of stacking generation—and their own feature importances—conferred predictive value.

**Figure 14:**
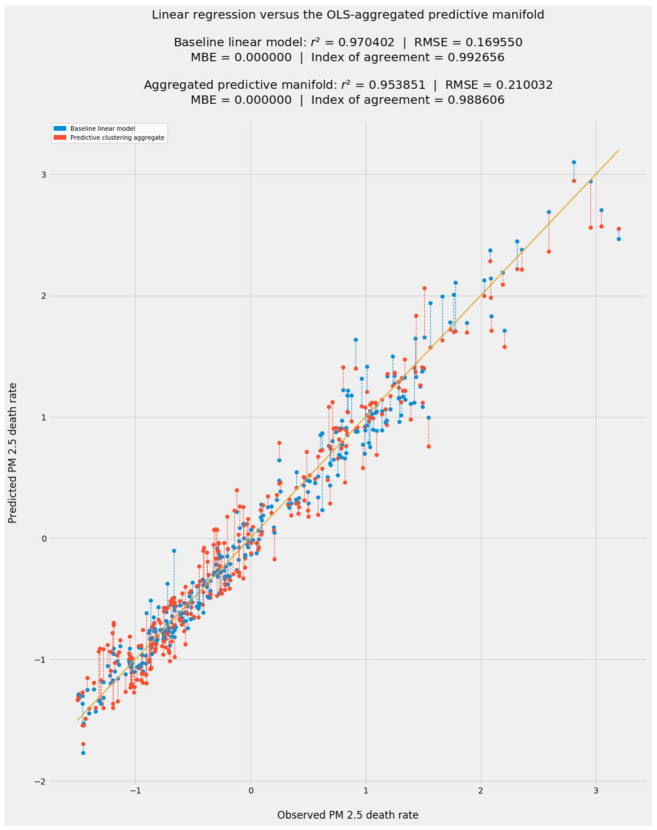
A “sundew plot” comparing conventional OLS with aggregated predictions through unsupervised machine learning.

The tentacles of the carnivorous sundew (*Drosera* *rotundifolia*) inspires the joint visualization of these two models. The “sundew plot” in Figure 14 combines scatterplots for two competing predictive models and uses vertical distances between each prediction (and relative to the ground truth) to compare the models’ accuracy.

Figure 14’s sundew plot shows the close visual relationship between OLS regression and the final, aggregated predictive manifold. Both are highly accurate representations of PM_2.5_ mortality rates. Although predictive unsupervised learning did not, in this instance, exceed the accuracy of more conventional methods, it validates the feature importances gathered through these steps:

- Actual feature importances from supervised machine learning models
- Emulated feature importances from linear models such as pooled OLS and all variants of fixed effects
- The weighting of actual and emulated feature importances according to the model-specific feature importances generated by the stacking blender

In other words, supervised learning does more than make predictions. It also preprocesses the data in advance of unsupervised clustering and manifold learning. In turn, unsupervised learning on the array of predictive variables, weighted according to the stacking blender’s evaluation of results from consciously predictive models, generates an independent set of predictions. Indeed, predictions from unsupervised machine learning cover the entirety of the dataset, without the splitting of data into training and test sets or expedients such as cross-validation. In datasets where this pipeline yields results that affirmatively outperform the OLS baseline, predictive uses of unsupervised learning demonstrate that all forms of machine learning can enhance conventional analysis.

### 3.7. The Environmental Kuznets Curve

So far we have emphasized the reduction of dimensionality from 23 predictive variables to a single dimension corresponding to the ground truth of premature mortality from PM_2.5_ pollution. Unsupervised learning can make a final contribution to the interpretation of PM_2.5_ mortality data.

Principal component analysis (PCA) can decompose 23 predictive variables into two cogent subcomponents, economic and health-based. This subsection explains the decomposition in detail and presents its results. Environmental Kuznets curves follow naturally from a plot of the ground truth of PM_2.5_ mortality against each composite index. Put simply, environmental Kuznets curves are a species of unsupervised machine learning.

#### 3.7.1. Extracting Economic and Health-Based Indexes through PCA

PCA can decompose a subset of independent variables in a high-dimensional dataset into a single, one-dimensional composite index. This is one of the simplest applications of unsupervised machine learning. Instead of covering the full 297 × 23 accuracy-weighted array, PCA can be applied to the appropriate slice of the data, as in a 297 × 11 subarray of economic variables or a 297 × 12 subarray of health-based variables. Aggregating those PCA results then enables a country-by-country comparison. Decomposition of an array variables provides a workable solution to the “composite index of environmental performance” often sought in the literature on environmental Kuznets curves [73] (p. 22).

The application of PCA to the relevant fraction of the posterior, accuracy-weighted array describes each country according to that set of independent variables. So applied, PCA can extracts a single index of economic indicators and a corresponding index of health-based indicators. Health-based indicators blend environmental and epidemiological factors that can be distinguished from possible economic drivers of public health.

PCA’s crudeness reveals the hidden strength beneath the environmental Kuznets curve. PCA offsets its mathematical simplicity by bringing interpretive clarity to machine learning’s black box. Like other efforts to reduce dimensionality, decomposition through PCA simultaneously simplifies complexity and clarifies understanding.

Among this article’s 23 predictive variables, 11 appear to be primarily economic: all three definitions of the poverty threshold, *emissions*, *real_GDP_pc*, *health_expenditures*, *environmental_taxes*, *social_contributions*, *spending*, *corruption*, and *gini*. Emissions per capita are a better indicator of economic activity than of public health, since they do not directly address exposure to PM_2.5_ pollution. The remaining 12 variables are primarily environmental or epidemiological: *expectancy*; *exposure*; and all indicators of incidence or mortality for cardiovascular disease, ischemic disease, COPD, asthma, and tracheal disease.

**Figure 15:**
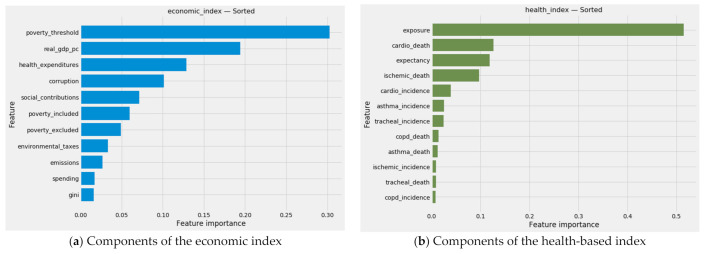
Individual components of the PCA-generated economic and health-based indexes: (**a**) the economic index; (**b**) the heath-based index.

Economic variables account for roughly 28.5 percent of feature weights in the stacking blender. Health-based variables account for the remaining 71.5 percent. The balance among components of each index is illustrated in Figure 15:

The economic index (Figure 15a) accords considerable weight to *real_gdp_pc* and *poverty_threshold*, a proxy for cost of living. Health-related expenditures and social contributions, both within the control of governments, are nontrivial components. Surprisingly, perhaps, so is *corruption*. That political trait accounts for a tenth of this index.

The health-based index (Figure 15b) is dominated by *exposure*, which accounts for more than half its weight. Three other variables—*cardio_death*, *expectancy*, and *ischemic_death*—each constitute roughly a tenth of the health-based index.

#### 3.7.2. The Economic Variant of the Environmental Kuznets Curve

Aligning either composite index with the vector of PM_2.5_ mortality rates instantly generates an environmental Kuznets curve. Table 6 reports the numerical results for the composite economic index. The application of PCA to the relevant slice of the posterior, accuracy-weighted PM_2.5_ dataset provides an independently credible ranking of EU-27 member states according to the cost of living, governmental exactions and expenditures (with varying degrees of relationship to public health), and GDP per capita.

Simple in absolute terms, Table 6 reports 27 instances with two values each. Plotting the PCA-generated economic index on the *x*-axis and PM_2.5_ mortality rates on the *y*-axis reveals a radically simplified and instantly interpretable environmental Kuznets curve.

**Figure 16:**
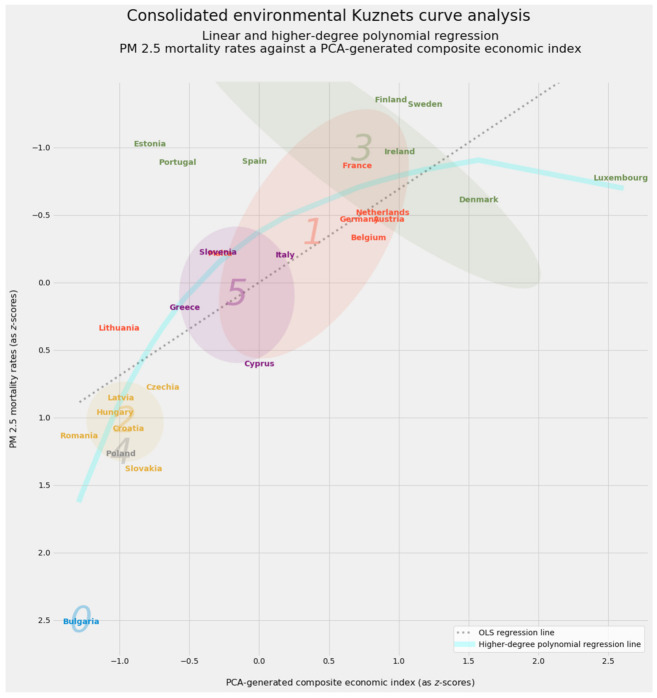
The environmental Kuznets curve for the PCA-generated composite economic index.

Figure 16 reports PM_2.5_ mortality on an inverted axis so that higher *z*-scores appear toward the bottom, as one would expect of bad health outcomes. Cluster membership is carried over from the posterior, accuracy-weighted analysis of data aggregated by country. The vertical and horizontal scales of the ellipses, as well as their tilt, indicate variability within each cluster along each of the chart’s two dimensions.

Higher-degree polynomial regression should reveal one of the three characteristic shapes of the environmental Kuznets curve (linear, inverted U, N-shaped). The unweighted average of quadratic, cubic, and quartic regression curves reveals the characteristic parabolic shape of an inverted U. In all events,, the downward curvature is very slight. This curve could be interpreted as a monotonically increasing environmental Kuznets curve.

The costlier, wealthier, and more generous countries of Denmark and Luxembourg have affirmatively lower levels of health than less wealthy but still relatively prosperous countries such as Finland, Sweden, Ireland, and France. The relatively poor countries of Spain, Portugal, and (perhaps most surprisingly) Estonia all achieved better health outcomes than Denmark and Luxembourg.

The shape and size of the ellipses characterizing each of the clusters also reveals information about each cohort. There are three visually distinct clumps:

- The healthy countries in clusters 3 and most of cluster 1
- An intermediate tier consisting of cluster 5 and two countries in cluster 1
- Clusters 2, 4, and 0, including impoverished and unhealthy Bulgaria on its own

The intermediate region covering clusters 5 and 0 reveal socioeconomic diversity. This region does not necessarily align with clusters found by unsupervised machine learning. This is particularly true along the horizontal, economic axis. EU-27 countries exhibit divergent public health outcomes relative to underlying economic conditions. The ellipse for cluster 1 tilts upward. The ellipse for the healthiest cluster, number 3, points downward. Indeed, cluster 3 is almost orthogonal to cluster 1. This suggests monotonic improvement in public health with increasing wealth—to a point. The downward slope of the ellipse for the healthiest countries is consistent with the inverted U interpretation.

The position of individual countries relative to the linear and polynomial regression curves also supports a clear interpretation. Positions above these curves, particularly the inverted parabola reported by higher-degree polynomial regression, indicate public health overperformance relative to the rest of Europe.

Positions below the curve indicate underperformance. Apart from the uniquely appalling case of Bulgaria, Cyprus may represent Europe’s greatest disappointment. Despite its relative prosperity, approximately 0.5*z* greater than its historical and cultural counterpart Greece, Cyprus underperforms expectations by nearly a full standard deviation.

The superlative performance of Estonia, Portugal, and Spain invites speculation about geospatial effects on PM_2.5_ mortality. These are not particularly prosperous countries. Indeed, all three have negative *z*-scores in the economic index. Estonia, in particular, shares a political history with the 10 formerly socialist countries at the bottom of the index. Yet all three boast one of the six lowest rates of PM_2.5_ mortality in the EU. Even Lithuania, otherwise mired with poorer countries in the east, was assigned to moderately healthy cluster 1, dominated by the original signatories of the Treaty of Rome.

Geography may have a profound impact. Estonia lies east of Sweden, a sparsely populated country with the second-best PM_2.5_ mortality rate. It is buffered by the Baltic Sea. Portugal and Spain occupy the Iberian peninsula. To their west lies the Atlantic Ocean. Indeed, Portugal achieves almost the same success as Spain in avoiding death from PM_2.5_ pollution, despite a markedly lower economic score. Isolation at the northern and western edges of Europe may explain these countries’ public health success. By contrast, the mostly landlocked Višegrad countries—Poland, Czechia, Slovakia, and Hungary—suffer cross-boundary pollution from wealthier neighbors to their west.

#### 3.7.3. A Health-Based Look at the Environmental Kuznets Curve

**Figure 17:**
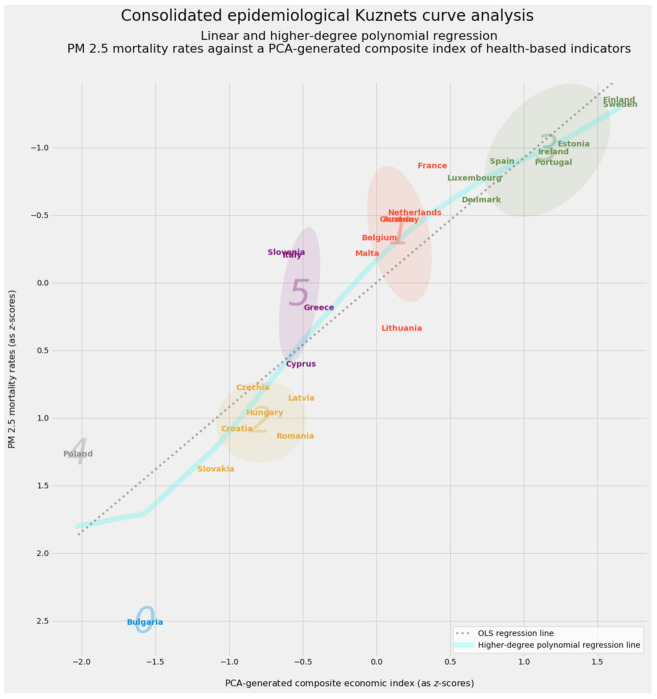
The environmental Kuznets curve for the PCA-generated composite health-based index.

Repeating the previous steps with the PCA-generated composite health-based index generates the curve in Figure 17. Unlike the economic variant of this curve, the health-based curve assumes a mostly linear form.

The health-based analysis should be wholly unsurprising, since it plots a public health outcome against a decomposed vector of health-based variables. Unlike the economic variant of this curve, however, Figure 17 cleanly distinguishes among the six clusters. Again, this comes as no surprise: the health-based index accounts for nearly three-fourths of the weight of the entire study.

The health-based curve explains many of the curiosities in the traditionally economic version of the environmental Kuznets curve. Lithuania aligns with the wealthier countries of cluster 1 because it stands on equal health-based footing. Though the Homeric cluster 5 lies not too far from Malta, there is a clear gap. Poland, despite death rates more in line with cluster 2, trails the rest of Europe so badly that it earns a cluster of its own. It may be worth asking why Poland outperforms its health-based indicators, while neighboring Lithuania, despite better health indicators and lower PM_2.5_-related mortality, lags the rest of cluster 1 and falls below the linear and polynomial regression lines.

**Figure 18:**
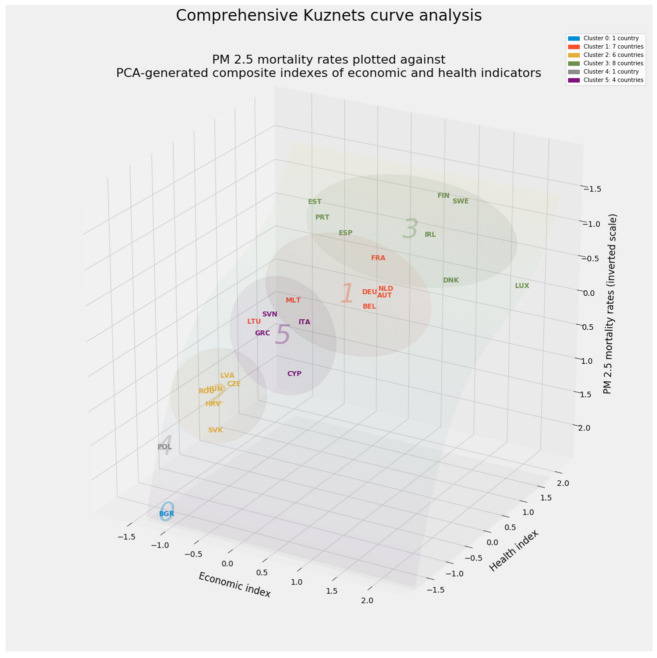
A comprehensive environmental Kuznets curve plotting PM_2.5_ mortality rates against PCA-generated composite economic and health-based indexes.

All that remains is to plot the economic variant of the environmental Kuznets curve against its health-based counterpart. In Figure 18, the mildly inverted parabolic shape of the environmental Kuznets curve based on the composite economic index contrasts with the monotonically increasing shape of the corresponding health-based curve.

The country-specific aggregation in Figure 18 captures this article’s qualitative essence. In a play on words combining political history with this study’s mathematical underpinnings, this radical reduction of information fulfills a manifold destiny.

## 4. Discussion

### 4.1. The Environmental Kuznets Curve: Bridging Quantitative Analysis with Policy Evaluation

#### 4.1.1. An Apt Metaphor for Unsupervised Machine Learning Writ Large

The formulation of environmental Kuznets curves connects the presentation of quantitative results with a policy-oriented discussion. Environmental Kuznets curves separate the complexities of PM_2.5_ pollution into distinct and tractable economic and health-based components. The qualitative elements of that summary of this article’s findings now facilitates a closer look at policy challenges and options within the European Union.

This study’s methodological pipeline included the use of unsupervised machine learning to cluster countries according to common features and their impact on the target variable of premature death from airborne PM_2.5_. Environmental Kuznets curves summarize but do not exhaust the broader descriptive and prescriptive goal of analyzing relationships among economic development, air quality, and human health.

Environmental Kuznets curves do excel in framing qualitative examination. Faced with overwhelming complexity, humans routinely adopt heuristic shortcuts. Among such expedients, environmental Kuznets curves boast the virtue of reflecting quantitative evidence, even if it has been condensed.

Economic development and transformation based on knowledge, innovations, new technologies, and environmental governance are driving improvements in environmental standards and quality [188,189,190,191,192,193]. These trends support a version of the environmental Kuznets curve that predicts superior environmental performance as an economy grows. Indeed, our environmental Kuznets curve analysis confirms work examining this relationship specifically within the European Union: “Eastern [European] countries appear to be performing generally quite well, … benefiting from EU membership and related policies in terms of environmental performance” [74] (p. 203).

Compressing 23 dimensions of data into one-dimensional, PCA-decomposed vectors condenses more than the data itself. The formation of these curves is an apt metaphor for unsupervised machine learning writ large. Manifold learning adds nothing to data; it extracts insights without regard to labels assigned by humans. If matrix operations improve the accuracy of predictive manifolds, that process validates the value of linear and supervised machine learning models that informed the calculation of vector weights.

The assignment of countries to separate classes primarily serves political convenience. In the absence of human supervision, clustering supplies labels and defines categories. Any distinction between economics and public health likewise hinges on human judgment. PCA and environmental Kuznets curves channel those judgments. The resulting categorical taxonomy, whether defined along geographic or policy-oriented boundaries, help human decision-makers orient themselves amid a sea of quantitative data.

#### 4.1.2. Closer Examination of Health-Based Factors

By the same token, the ability to summarize groups of variables as a single composite index, the heuristic shortcut that PCA and the environmental Kuznets curve enable, cannot and should not take the place of closer analysis. In particular, health-based factors warrant deeper research, especially on a country-by-country basis.

The *exposure* and *expectancy* factors exerted great influence on the health-based index. *Exposure* alone accounted for more than half the weight of the index. As a group, variables reporting incidence and mortality rates for five types of disease held less sway. Within the aggregated vector of feature importances, only two of those ten variables—*cardio_death* and *ischemic_death*—exceeded their expected 4.35 percent share of feature weights. Coronary diseases carried more weight than respiratory diseases. Indeed, all indicators of respiratory disease (asthma, COPD, and the trio of tracheal, bronchial, and lung cancers) totaled less than 10 percent of the vector of aggregated feature weights.

In the absence of clear, let alone decisive, machine-language results, traditional statistical tools may shed greater light. As described in Table 1, most linear models treated *ischemic_death* as a statistically significant variable. Half of the linear models did the same for *cardio_death* correlation to premature deaths from PM_2.5_ pollution. As a rule, however, the FEE and IV2SLS models support few causal inferences from strictly epidemiological features.

Although evidence from this study is far from exhaustive, there are suggestions that PM_2.5_ mortality shows (considerably) greater connection to cardiovascular and ischemic heart disease than to any respiratory disorder. This inference may be slightly counterintuitive, since PM_2.5_ enters the body through the lung. The weight of the evidence, however limited, does suggest that national governments and the EU as a whole should focus public health efforts on coronary rather than respiratory disorders—to the extent that the components of the cardiopulmonary system are truly distinct. At a minimum, health-based expenditures, the third largest component of the composite economic index depicted in Figure 15a, might target cardiovascular and ischemic disease ahead of asthma, COPD, and cancers of the trachea, bronchus, and lung.

The balance of this discussion exploits insights drawn from the environmental Kuznets curves and other exercises in unsupervised learning. Section 4.2 examines challenges facing individual EU member states. It relies upon the clusters of countries revealed through Section 3.5’s application of unsupervised learning to country-level aggregations of PM_2.5_ data and depicted in Figure 8 and Figure 9. Section 4.2 also draws upon the aggregated vector of feature importances generated by the stacking blender and depicted in Figure 7. All normative discussion in this section explicitly evaluates policies that either have been adopted by the European Union or may arise as options for the future.

### 4.2. Cluster- and Country-Specific Analysis of Individual EU-27 Member States

Clustering analysis clarifies the extent to which the European Union has harmonized its response to PM_2.5_ pollution. This research can also inform public health and macroeconomic forecasting and facilitate the diffusion of best practices throughout the EU-27.

This discussion refers throughout to clusters 0 through 5 of EU-27 member states as depicted in Figure 9. Clusters 3 (Denmark, Estonia, Finland, Ireland, Luxembourg, Portugal, Spain, and Sweden) and 1 (Austria, Belgium, France, Germany, Malta, and the Netherlands) have much lower rates of PM_2.5_ morality than clusters 2 (Croatia, Czechia, Hungary, Latvia, Romania, and Slovakia), 0 (Bulgaria), and 4 (Poland). Cluster 5 (Cyprus, Greece, Italy, and Slovenia) occupies an intermediate position. The convergence of environmental, socioeconomic, and public health factors by cluster draws attention to differences among clusters. Differences within clusters are also worthy of deeper evaluation.

In addition to the obvious west–east divide between the low- and high-mortality clusters, an arguably subtler demographic distinction lurks. Specifically, clusters 3 and 1 have a significantly higher number of inhabitants. Those two low-mortality clusters contain 62 percent of the population of the EU-27. Cluster 1 contains 43 percent of the EU-27′s total population; cluster 3 contains 19 percent.

*Emissions* and *gini* do not figure prominently in this study’s aggregated vector of feature importances. Each of those variables accounts for less than 1 percent of the overall vector, far short of the presumptive 4.35 percent that would be assigned to each feature in an equally weighted vector containing 23 features. At 5.53 percent, more than its proportional share of feature importances, *real_gdp_pc* is a non-negligible feature. Nevertheless, that feature does not seem to capture the full effect of inequality upon PM_2.5_ mortality.

At 8.63 percent, *poverty_threshold* secures more than its share of weight within the aggregated vector. That variable satisfies traditional tests of statistical significance within some linear models. Because *poverty_threshold* indirectly measures productivity and cost of living, that variable may capture elements of inequality that eluded other predictors.

All three poverty indicators represent this study’s indicators of social status. Socially deprived communities in poorer regions are especially vulnerable to the harmful effects of PM_2.5_ pollution [6]. At the other extreme of the distribution, the poverty threshold affects Luxembourg’s almost outlandish place in the environmental Kuznets curves. Luxemburg is the richest country in the EU-27 and the second richest in the world.

Even without direct evidence that PM_2.5_ emissions are linked to higher mortality, lower-mortality countries might monitor PM_2.5_ emissions from household, industrial, and transportation-related activities. Economic activity and population density may be the latent and true factors. Emissions from residential heating, together with road traffic and industrial emissions, expose many people to PM_2.5_ pollution [6]. All are urban activities characterizing the wealthier, more densely populated countries in clusters 3 and 1.

Low population density carries its own set of possible policy prescriptions. PM_2.5_ pollution originates in many economic sectors, such as energy, transport, and agriculture [194]. These activities vary widely by country. For example, in Estonia, Malta, and Portugal airborne PM_2.5_ stems mostly from intensive transportation. Agriculture makes significant contributions to PM_2.5_ air pollution in Croatia and Germany [194].

Within the aggregated vector of feature importances, *exposure* outweighs *emissions* by nearly a multiple of 50. PM_2.5_ exposure does vary considerably according to physical and urban geography. Again, cluster 1 hosts more than two-fifths of the EU-27 population and includes the Union’s two most populous countries, Germany and France.

Malta also belongs to cluster 1. This small southern island state has a total area of 246 km^2^ and a population density exceeding 1500 inhabitants per km^2^. The EU-27 average is 109 inhabitants per km^2^. Since exposure to PM_2.5_ pollution is generally based on emissions and the number of inhabitants at the national level, the relationship between these variables is clear.

Country-by-country evaluation of PM_2.5_ emissions and exposure has obvious limits. This study took no account of cross-border transport of PM_2.5_ or its impact on other variables. Future research might evaluate cross-border air pollution through additional geospatial features. The most nuanced work on the environmental Kuznets curve counsels closer attention to relationships between economic and geospatial factors [55]. At a bare minimum, the superlative performance of Nordic and Iberian countries, extending as far as the Baltic states, deserves much closer examination.

For now, however, there are two additional sources of country-specific information. Fixed effects linear models break out entity, time, or other categorical effects and convert them to dummy variables. The coefficients on entity dummies in the FEE and FETE models yield insights into differences among individual countries (Table 7).

As a rule, wealthier, cleaner countries tended to register negative coefficients, while the highest positive coefficients applied to the most mortality-stricken countries of eastern and southeastern Europe. This is especially true of the FETE model. Lithuania is a salient exception. Its negative coefficient in both FEE and FETE suggests that its placement among the wealthier, cleaner countries of cluster 1 might not have been a complete fluke.

At 8.48 percent, life expectancy at birth for women and men carries almost double its proportional 4.35 percent of the weight among aggregated feature importances. Low-mortality cluster 3 is home to the longest-lived citizens of the EU-27: citizens of Spain live an average of 83.43 years. Although longevity has many mixed implications for government and private spending on pensions and health care, and more generally for economic growth and welfare, the wealthier countries in clusters 3 and 1 are enjoying greater success in meeting these demographic challenges.

Bulgaria, the lone country in cluster 0, has the European Union’s lowest life expectancy (61.25 years). Life expectancy at birth is influenced not only by environmental quality and socioeconomic status but also by behavioral patterns and individual predispositions. Lower life expectancy throughout clusters 0, 2, and 4 may be more directly connected to premature mortality caused by airborne PM_2.5_. National policies in these poorer, less healthy countries should strive to improve the well-being of citizens. Improvement in overall life expectancy should reduce premature PM_2.5_ mortality.

Well-harmonized public health and environmental measures, supplemented with an improvement in the status of the elderly, could yield substantial benefits. All southeastern countries (Bulgaria, Croatia, Greece, Romania) and six central countries (Czechia, Slovakia, Hungary, Poland, Latvia, Lithuania) could reduce PM_2.5_ mortality through effective social efforts to enhance the well-being of their elderly populations.

As much as wealthier countries have lagged in their transition toward a “cleaner and greener economy,” poorer countries need an even faster transition, with more extensive EU support. Poorer countries should also consider cleaner public transport or congestion charges, cleaner fuels for heating, and more stringent industrial pollution controls.

The wealthier countries in clusters 3 and 1 have a longer tradition of continuous economic progress, environmentally friendly innovations and technologies, and more advanced environmental management. Consequently, those counties enjoy superior environmental performance and better public health.

Richer countries nevertheless face pronounced challenges. Despite innovative triumphs and robust industrial performance, these wealthy countries still rely heavily on fossil fuels, face very dense urban and suburban traffic, and host large populations. The failure of the richest countries, especially Denmark and Luxembourg, to translate their wealth into superlative management of PM_2.5_ mortality suggests declining returns from growth, knowledge, and innovation.

This study has exposed inequality in health among EU-27 countries. At the same time, those inequalities stem from complex and highly localized considerations that belie the superficial cohesion of machine-discovered clusters or even the countries within them. In Bulgaria (cluster 0), for example, residential heating with wood and coal predominates and is the most significant source of PM_2.5_ [195]. Germany (cluster 1) and Poland (cluster 4) are Europe’s largest coal producers, but it is obvious that Germany fights air pollution and premature PM_2.5_ mortality more effectively.

Cyprus’s singular underperformance warrants close scrutiny. Cyprus is located at the extreme southeastern edge of the European Union. No other member of cluster 5 suffers as much premature PM_2.5_ mortality. Even Lithuania, the most prominent outlier among the richer countries of cluster 1, outperforms Cyprus, despite the Baltic state’s much deeper poverty and traumatic 20th-century history.

Underperformance in Cyprus might inform policy there and elsewhere in Europe. Cyprus is unique among EU-27 member states not merely because of its location at the southeastern corner of the union. Its topography and blend of emission sources undoubtedly affect its PM_2.5_ mortality rate. Transport and residential heating rank as Europe’s leading sources of air pollution [6]. Other sectors contributing to air pollution (in declining order) include energy production and distribution, manufacturing and extractive industry, agriculture, and waste (including wastewater management). Terrestrial transport and home heating deposit particulate matter close to the soil, precisely where humans are likeliest to encounter pollution.

The Mediterranean climate of Cyprus, with hot, dry summers and mild winters, virtually eliminates the need for residential heating. On the other hand, tourist traffic raises the contribution of surface transportation to pollution in Cyprus, especially on a seasonal basis. Without further evidence, this study cannot draw definitive conclusions. Departures from the model’s predictions, however, identify opportunities for closer study and, ideally, the crafting of policies to suit the diverse members of the European Union.

### 4.3. Policymaking at the European Level: Implications and Recommendations

Preventable loss of life and well-being to PM_2.5_ pollution poses a global and uniquely anthropocentric challenge. Around the world, and not just in Europe, PM_2.5_ pollution arises from patterns of human behavior in production, energy consumption, food, and mobility. Its toll will grow as populations around the world continue to age.

PM_2.5_ pollution is a primarily urban phenomenon, since sources of PM_2.5_ are located mainly in cities. As urban populations rise, the road network will grow to meet their demand. Consequently, surface transport as a major source of PM_2.5_ will also grow, emitting particulates at the level where humans breathe.

By the same token, wealthier countries have resources to reduce emissions and exposure. At the multinational, national, and local levels, governments can implement measures such as surcharges on personal vehicles that enter city centers. Subsidies for electric and hybrid vehicles can also lower the PM_2.5_ footprint of urban life. The greening of transportation and energy systems can dramatically improve public health. Its economic impact also promises to be considerable, from a reduction of the negative effects of PM_2.5_ and related medical costs to increased labor force productivity.

As one of the world’s most rapidly aging regions, Europe is peculiarly susceptible to the negative effects of PM_2.5_ pollution. Europe’s geographic, socioeconomic, and political diversity, however, makes this public health problem exceptionally challenging. Elderly people are often socioeconomically isolated and health-compromised. Individual EU-27 member states should consider radical and rapid changes in policy, with respect not only to air quality but also to socioeconomic factors.

The possibility of policymaking discretion at the EU level invites distinct normative considerations. All EU-27 countries continue to face significant environmental and public health challenges from PM_2.5_ pollution. Cross-border pollution has proved especially intractable. The European Union’s Ambient Air Quality Directive set the limit for annual average PM_2.5_ levels at 25 μg/m^3^ [10]. The stricter value of the WHO air quality guidelines for annual average PM_2.5_ levels is 10 μg/m^3^ [9,196]. EU-27 countries satisfied the European limit in 2018, but some exceeded WHO’s 10 μg/m^3^ guideline [197].

According to the European Environmental Agency, the last 50 years of environmental policymaking within the European Union have curbed air pollution [6]. The EU-27, however, has failed to eliminate PM_2.5_ pollution as a significant influence on human health. Policies implemented to reduce PM_2.5_ emissions from road transport, domestic heating, industry, and agriculture have proved insufficient.

Table 8 provides quantitative evidence that at least the linear FETE model’s estimated time effects show a progressive trend toward lower PM_2.5_ mortality from 2008 to 2011.

The COVID-19 pandemic has highlighted the need for a common European response to public health and economic challenges. Lockdown measures have significantly reduced PM_2.5_ emissions [6]. Narrowly “siloed” approaches to public health have not yielded significant progress. They have done little beyond maintain the partially satisfactory status quo. European citizens are certainly less than fully satisfied. More than half of the respondents to the 2019 Eurobarometer survey think that public authorities, car manufacturers, energy producers, and even individual households are not doing enough to improve air quality [198].

The possibility of more radical change remains open. The EU-27 has announced a paradigm of sustainability transitions based on decoupling of harmful environmental pollution from economic growth [199]. This ambition is embodied in new policies and initiatives such as the European Green Deal [200]. These measures and policies suggest that economic growth does not have to come at the expense of environmental quality [73] (p. 22).

A more tangible step involves the strengthening of European air quality standards. To improve air quality and public health, the European Commission is preparing to align the EU’s air quality standards more closely with WHO’s stricter emission limits. The May 2021 proposal to lower European limits on PM_2.5_ emissions is the centerpiece of an EU Action Plan designed to tackle air pollution at its sources, from transport, buildings, and energy consumption to industry and agriculture [201].

Human suffering and economic loss from PM_2.5_ pollution force a difficult question: Are EU-27 public health and environmental policies efficient and harmonized? Although most EU-27 countries have achieved significant reductions in PM_2.5_ emissions [6], significant impacts on morbidity and mortality persist [14,15,16].

Reduced PM_2.5_ emissions are insufficient on their own to significantly reduce adverse health effects. Indeed, that factor lacked statistical significance or machine learning feature weight in all models. Other socioeconomic improvements should lower losses to PM_2.5_ pollution. A leading priority should be improving the quality of health care.

Consistent with Europe’s longstanding aspiration toward ever closer union, EU-27 countries should strive to bridge their historical, cultural, and political gaps. Persistent health losses traceable to PM_2.5_ pollution highlight the urgency of such a transition. A more efficient and comprehensive public health policy should complement and strengthen the EU-27′s common environmental and market policies. Public health shortcomings highlight a particular vulnerability in the political and legal foundations of European integration: health policy and economic development are left to individual member states.

PM_2.5_ pollution remains a public health challenge of the highest order. Striving to “show you something different from … your shadow at evening,” this article set out to “show you fear in a handful of dust” [202] (pp. 31–32). The challenge remains urgent. For “[w]e who were living are now dying/With a little patience” [202] (p. 60).

## 5. Conclusions

This article identified 23 possible variables affecting the complex phenomenon of premature death from PM_2.5_ pollution. The data’s high level of dimensionality demanded—and received—a comprehensive analytical pipeline:

- Conventional linear models
  ○ Pooled OLS
  ○ Fixed effect models
  ○ Random effects
  ○ Instrumental value, two-stage least squares
- Supervised machine learning alternatives to linear regression
  ○ Decision tree ensembles such as random forests and extra trees
  ○ Boosting models such as AdaBoost, XGBoost, and LightGBM
- Stacking generalization
  ○ As an aggregator of predictions
  ○ As an aggregator of real and emulated feature importances to advance interpretation and causal inference
- Unsupervised machine learning
  ○ Clustering
  ○ Manifold learning
  ○ A suite of unsupervised methods leading to predictive manifolds
- Environmental Kuznets curves

This article has made scientific contributions at two levels. Beyond adding insight and nuance to knowledge about PM_2.5_ pollution and European Union policymaking, this article has devised a set of conventional and machine learning methods that can be generalized to other disciplines.

The methodological tools deployed in this article provide a blueprint for future work. Although the environmental Kuznets curves distinguished between economic and health-based variables within the full set of 23 predictors, that analytical method applies to any experimental design whose variables can be meaningfully distinguished along qualitative lines. Neither the elaborate extraction of an aggregated vector of feature importances through stacking generalization nor the simple decomposition of any arbitrary slice of that vector through PCA is limited by scientific subject matter.

Ample room remains for further research. This article revealed but could not conclusively resolve country-specific differences within the EU-27. Future work could explore geospatial effects. Cross-boundary effects remain one of the most important and complex components of all pollution problems. Proximity to sea and ocean water palpably affects PM_2.5_ pollution and its impact on human health. Whether the influence of water extends beyond the absence of human activity remains a mystery. European countries also differ markedly in their mix of economic activities, ranging from transportation, energy production, and residential heating to manufacturing, agriculture, and extractive industries. The intensity of pollution from each sector also differs by country.

This article also detected social and political differences traceable to various experiments with socialism and central planning in recent European history. Some models, at least, did not wholly dismiss possible contributions from more abstract economic and political phenomena expressed through the Gini coefficient of income inequality or a corruption index. There may be effects beyond those expressed through the poverty threshold as a proxy for a country’s cost of living and distribution of wealth and earning power.

The European Union is renowned for its diversity. Its celebrated linguistic and cultural differences transcend those of other large federations with comparable or larger geographic footprints, such as Russia, China, Australia, Canada, and the United States. Indeed, several of the largest member states of the EU-27 reflect that diversity on a subnational basis. Local independence, after all, has marked the history of Germany, Italy, Spain, and Belgium. A better level of data granularity for further research within Europe may therefore be NUTS-2 or even NUTS-3 rather than the national level.

As a technical matter, research into differences between geographic locations at any political or topographical level would benefit from further refinement in machine learning. This article’s analytical pipeline is much more effective in aggregating predictions and interpretive indicators than in attributing contributions from each predictor at a country-specific level. As enticing as the prospect may be, this experimental design cannot yet conduct sensitivity analysis according to each geographic entity or each cluster uncovered by unsupervised machine learning.

## Figures and Tables

**Table 1:** Parameters for all linear models (*a*). See notes below.

Variable	Pooled OLS	FEE	FTE	FETE	RE (*b*)	IV2SLS (*c*)
*expectancy*	−0.228050 ***	−0.679440 ***	−0.223873 ***	−0.333790 **	−0.345283 ***	0.443984 ***
*poverty_threshold*	0.151001 ^†^	0.259350 **	0.265349 **	0.129913	0.110895	0.362384 **
*poverty_excluded*	−0.111437 ***	−0.050287	−0.122527 ***	−0.037093	−0.115381 ***	0.074701
*poverty_included*	0.043042	0.098076 ***	0.062356 *	0.108652 ***	0.063563 *	−0.192787 ***
*emissions*	−0.006356	0.019317	0.000065	0.023919	−0.011046	0.078672 **
*exposure*	0.425233 ***	0.219233 ***	0.414018 ***	0.128386 ***	0.339615 ***	1.476388 ***
*cardio_incidence*	−0.256236 ***	0.064386	−0.273505 ***	−0.143618	−0.199337 ***	−0.031434
*ischemic_incidence*	0.001203	0.009228	−0.005284	−0.002822	0.006475	0.021552
*copd_incidence*	0.014185	0.001154	0.012108	0.000839	0.001079	−0.027896
*asthma_incidence*	0.000686	0.063843	−0.010856	−0.012070	0.074828 *	−0.039956
*tracheal_incidence*	0.114423 ***	0.126039 *	0.115269 ***	0.151985 **	0.165075 ***	−0.068278
*cardio_death*	0.311744 ***	0.037946	0.326050 ***	0.024614	0.119257 *	0.096857
*ischemic_death*	0.309552 ***	0.344225 ***	0.314529 ***	0.336414 ***	0.397895 ***	−0.040304
*copd_death*	0.017858	−0.002824	0.012587	−0.003727	0.002650	0.025019
*asthma_death*	0.012423	−0.010763	0.008116	−0.009243	−0.000844	0.066837 **
*tracheal_death*	0.003558	0.005269	0.005872	−0.002371	0.011968	0.020837
*real_gdp_pc*	−0.091075	0.014715	−0.130006 *	0.172788 ^†^	−0.098530	0.012191
*health_expenditures*	−0.079581	0.059845	−0.139424 *	−0.032978	−0.047508	−0.304068 ***
*environmental_taxes*	−0.026935	−0.012153	−0.020793	0.056189 *	−0.046456 *	−0.188568 ***
*social_contributions*	−0.067829 ***	−0.222701 ***	−0.071533 ***	−0.269718 ***	−0.062536 *	−0.133907 ***
*spending*	−0.006306	−0.052013 ^†^	0.016360	0.000347	−0.020006	−0.022764
*corruption*	−0.045392 **	−0.013054	−0.084725	0.175724 ***	−0.031020 *	−0.060887 **
*gini*	0.006347	−0.002253	−0.000984	0.017345	0.012945	−0.094555 **

Notes to Table 1: Asterisks and plus signs indicate statistical significance at these levels: ***: *p* < 0.001; **: *p* < 0.01; *: *p* < 0.05; †: *p* < 0.1. The random effects model alone has a nonzero constant: 0.001197. That constant is ignored in subsequent efforts to harmonize linear models with machine learning. The parameter for *exposure* in IV2SLS refers to the estimation of endogenous variable *welfare_25* through *exposure* as an instrument. Although this coefficient is larger in absolute terms than any other in Table 1, it reflects an across-the-board scaling of all IV2SLS parameters relative to those of other linear models.

**Table 2:** Feature importances for all supervised machine learning models.

Variable	Random Forest	Extra Trees	AdaBoost	Gradient Boosting	XGBoost	LightGBM
*expectancy*	0.052014	0.098962	0.082059	0.061310	0.075148	0.068000
*poverty_threshold*	0.161033	0.084122	0.212715	0.001461	0.055871	0.050000
*poverty_excluded*	0.004967	0.005565	0.003094	0.003471	0.000797	0.012000
*poverty_included*	0.004442	0.003075	0.003895	0.001881	0.003689	0.056000
*emissions*	0.002551	0.003434	0.000456	0.000959	0.003930	0.036000
*exposure*	0.413803	0.339363	0.459060	0.717532	0.406206	0.160000
*cardio_incidence*	0.007967	0.018523	0.001447	0.003704	0.006088	0.030000
*ischemic_incidence*	0.011549	0.009752	0.001243	0.000447	0.002240	0.022000
*copd_incidence*	0.003862	0.003532	0.000336	0.000258	0.004328	0.028000
*asthma_incidence*	0.022526	0.018407	0.019316	0.008589	0.019928	0.038000
*tracheal_incidence*	0.003572	0.007471	0.000323	0.000678	0.002552	0.032000
*cardio_death*	0.083091	0.110241	0.030849	0.107699	0.139429	0.072000
*ischemic_death*	0.075020	0.114351	0.015745	0.014557	0.041431	0.070000
*copd_death*	0.002787	0.005753	0.002103	0.000533	0.005204	0.054000
*asthma_death*	0.008979	0.008653	0.001088	0.000058	0.006329	0.036000
*tracheal_death*	0.002632	0.004054	0.001344	0.000566	0.002391	0.034000
*real_gdp_pc*	0.062514	0.054825	0.128733	0.004961	0.107755	0.032000
*health_expenditures*	0.035335	0.063784	0.022096	0.026985	0.037256	0.026000
*environmental_taxes*	0.002992	0.003486	0.000781	0.002747	0.003695	0.020000
*social_contributions*	0.011451	0.005228	0.001485	0.028149	0.001857	0.044000
*spending*	0.001692	0.002186	0.000382	0.001251	0.001980	0.026000
*corruption*	0.022396	0.032298	0.009096	0.011921	0.071043	0.040000
*gini*	0.002823	0.002935	0.002354	0.000285	0.000853	0.014000

**Table 3:** Indexes of diversity and concentration for machine learning models’ feature importances.

Diversity or Concentration Index	Random Forest	Extra Trees	AdaBoost	Gradient Boosting	XGBoost	LightGBM
Gini coefficient	0.733565	0.687512	0.825314	0.879177	0.745226	0.313739
Simpson’s index	0.219090	0.166459	0.281478	0.532226	0.213564	0.063896
1/Simpson	4.564335	6.007497	3.552676	1.878900	4.682437	15.650432

**Table 4:** Emulated feature importances for all linear models.

Variable	Pooled OLS	FEE	FTE	FETE	RE	IV2SLS
*expectancy*	0.103498	0.315691	0.088595	0.170067	0.161772	0.120627
*poverty_threshold*	0.056767	0.119627	0.103650	0.044483	0.033923	0.098100
*poverty_excluded*	0.050569	0.018111	0.048487	0.012039	0.054032	0.016395
*poverty_included*	0.015629	0.045555	0.023372	0.055605	0.028999	0.052377
*emissions*	0.000249	0.004559	0.000000	0.009023	0.001037	0.021324
*exposure*	0.192986	0.101863	0.163842	0.065685	0.159116	0.401122
*cardio_incidence*	0.116289	0.009015	0.108236	0.056032	0.093379	0.001261
*ischemic_incidence*	0.000003	0.002564	0.000276	0.000140	0.000789	0.003647
*copd_incidence*	0.003198	0.000007	0.001871	0.000003	0.000004	0.005671
*asthma_incidence*	0.000000	0.017970	0.000483	0.000233	0.034137	0.006785
*tracheal_incidence*	0.051929	0.055625	0.045616	0.077112	0.077341	0.014908
*cardio_death*	0.141481	0.006410	0.129030	0.002597	0.053801	0.016227
*ischemic_death*	0.140486	0.159939	0.124470	0.172176	0.186422	0.001870
*copd_death*	0.005238	0.000092	0.001989	0.000269	0.000050	0.004565
*asthma_death*	0.001795	0.001808	0.000476	0.001564	0.000001	0.018044
*tracheal_death*	0.000072	0.000545	0.000264	0.000073	0.003248	0.003229
*real_gdp_pc*	0.032866	0.000087	0.048509	0.072957	0.036958	0.000049
*health_expenditures*	0.025815	0.014293	0.052561	0.003200	0.008734	0.082605
*environmental_taxes*	0.009734	0.000703	0.004754	0.026837	0.020746	0.051232
*social_contributions*	0.030772	0.102449	0.028303	0.137955	0.027745	0.036381
*spending*	0.000121	0.021325	0.001426	0.000000	0.003342	0.001610
*corruption*	0.020329	0.001757	0.023788	0.089896	0.013509	0.016367
*gini*	0.000175	0.000004	0.000001	0.002056	0.000916	0.025607

Abbreviations—Pooled OLS: pooled ordinary least squares; FEE: fixed entity effects; FTE: fixed time effects; FETE: fixed entity and time effects; RE: random effects: IV2SLS: instrumental value/two-stage least squares.

**Table 5:** Indexes of diversity and concentration for linear models’ emulated feature importances.

Diversity or Concentration Index	Pooled OLS	FEE	FTE	FETE	RE	IV2SLS
Gini coefficient	0.640728	0.729023	0.608118	0.636847	0.626618	0.698973
Simpson’s index	0.113195	0.167060	0.100787	0.110444	0.112763	0.201177
1/Simpson	8.834318	5.985860	9.921939	9.054360	8.868171	4.970742

**Table 6:** The environmental Kuznets curve for the PCA-generated composite economic index.

Country	Economic Index	PM_2.5_ Mortality
Romania	−1.285505	1.134495
Bulgaria	−1.273869	2.512904
Hungary	−1.029198	0.961135
Lithuania	−1.000805	0.338977
Latvia	−0.998795	0.857050
Poland	−0.987242	1.269684
Croatia	−0.936146	1.080966
Slovakia	−0.825427	1.378815
Estonia	−0.779594	−1.025214
Czechia	−0.692113	0.775506
Portugal	−0.581820	−0.887409
Greece	−0.533010	0.186775
Slovenia	−0.293839	−0.220876
Malta	−0.276277	−0.212840
Spain	−0.031286	−0.894877
Cyprus	0.001373	0.602726
Italy	0.187608	−0.201211
France	0.704757	−0.861699
Germany	0.716147	−0.465943
Belgium	0.784445	−0.330687
Netherlands	0.886835	−0.516262
Austria	0.931414	−0.465291
Finland	0.944055	−1.349146
Ireland	1.006518	−0.965832
Sweden	1.188469	−1.319755
Denmark	1.572643	−0.611526
Luxembourg	2.590962	−0.770464

**Table 7:** Estimated entity effects for the FEE and FETE models.

Country	FEE	FETE
Austria	0.014747	−0.045943
Belgium	0.243024	0.186347
Bulgaria	−1.163098	0.964617
Croatia	0.554713	0.850505
Cyprus	0.661399	0.455677
Czechia	0.743081	0.943984
Denmark	−1.195171	−1.663294
Estonia	−0.870172	−0.544080
Finland	−0.796109	−0.961272
France	0.267742	0.077985
Germany	0.054640	−0.036524
Greece	0.555249	0.222769
Hungary	0.016704	0.438898
Ireland	−0.668040	−1.269296
Italy	0.610340	0.378435
Latvia	−0.278896	0.464102
Lithuania	−0.468829	−0.034647
Luxembourg	−0.561520	−1.175075
Malta	0.477462	0.071157
Netherlands	0.167337	−0.258295
Poland	0.654021	0.937754
Portugal	0.322213	−0.133454
Romania	0.249310	0.822802
Slovakia	0.746669	1.303252
Slovenia	0.544619	0.490787
Spain	0.514152	0.007326
Sweden	−0.979204	−1.297142

**Table 8:** Estimated time effects for the FETE model.

Year	FETE
2008	0.399014
2009	0.392836
2010	0.421352
2011	0.426721
2012	−0.052770
2013	−0.227695
2014	−0.193217
2015	−0.328103
2016	−0.260225
2017	−0.338013
2018	−0.298708

**Figure A1:**
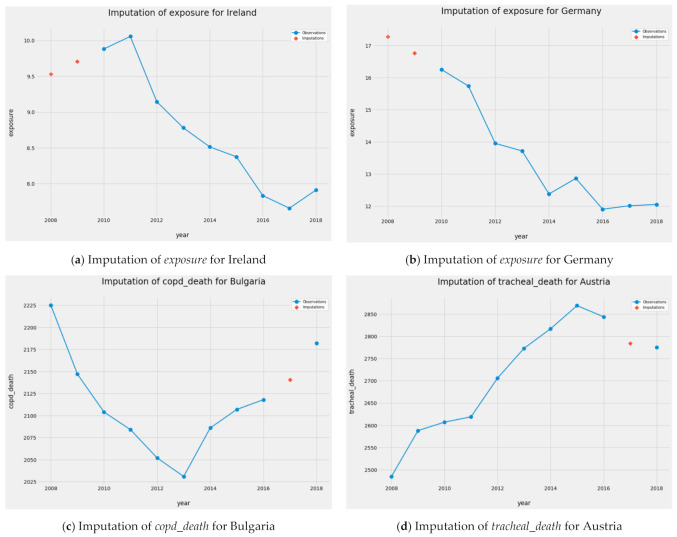
Imputing missing values for *exposure*, *copd_death*, and *tracheal_death* using LOESS and linear or cubic splines.

**Figure A2:**
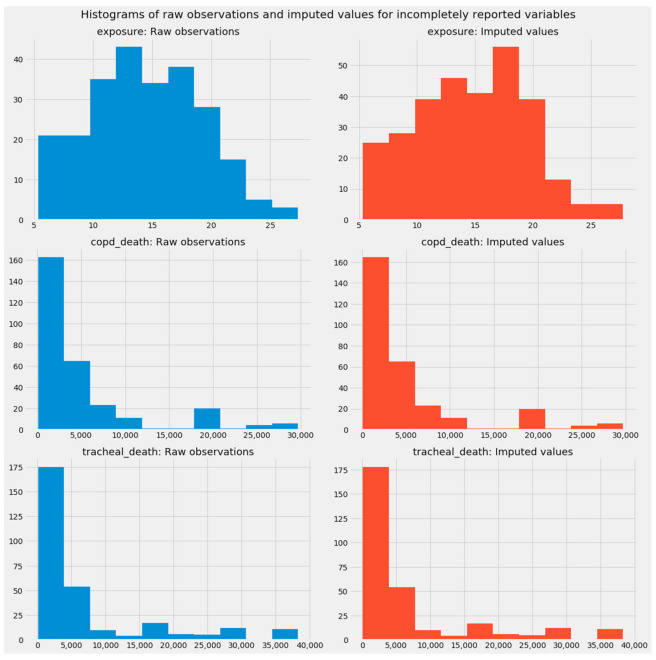
A before-and-after comparison of imputed data series: Histograms of raw observations and imputed values for incompletely reported variables.

## Data Availability

Data is available from the authors and will be posted upon publication of this article.

## Appendix A

The dataset contains a single dependent variable.

0. Premature death rates attributable to PM_2.5_ pollution: This data appears in the OECD repository, “Mortality, Morbidity and Welfare Cost from Exposure to Environment-Related Risks.” Data for 2018 is derived from “Premature death rates attributable to outdoor air pollution (PM_2.5_); Crude death rate per 100,000 population.” Data for 2008 through 2017 is derived from “Premature deaths from ambient particulate matter for persons more than 64 years old, both sexes.”

OECD, https://stats.oecd.org/viewhtml.aspx?datasetcode=EXP_MORSC&lang=en (accessed on 12 March 2021).

The dataset contains 23 independent variables:

1. Life expectancy at birth (in years) is the average number of years a newborn is expected to live if mortality patterns at the time of its birth remain constant in the future. It reflects the overall mortality level of a population, and summarizes the mortality pattern that prevails across all age groups in a given year. It is calculated in a period life table that provides a snapshot of a population’s mortality pattern at a given time.

World Bank, https://blogs.worldbank.org/opendata/what-does-life-expectancy-birth-really-mean (accessed on 12 March 2021).

2. The threshold at which a single person is at risk of poverty (in euros) is determined by calculating the equivalized income per household member for all households. Afterwards, the middle value (the median) of the income distribution is determined and 60 percent of the median is determined as the risk-of-poverty threshold. Everyone with the income below the threshold is in a worse situation than others, but they do not necessarily live in deprivation. The at-risk-of-poverty threshold is presented in currencies, while the at-risk-of-poverty rate is presented in relative terms as a percentage.

Eurostat, https://ec.europa.eu/eurostat/statisticsexplained/index.php?title=Glossary:At-risk-of-poverty_rate (accessed on 12 March 2021).

3. The rate of risk from poverty *before* social transfers (with pensions *excluded* from the definition of social transfers) is calculated as the percentage of people (or thousands of people) who are at risk of poverty, based on the equivalized disposable income before all social transfers—excluding pensions, over the total population.

Eurostat, https://ec.europa.eu/eurostat/statisticsexplained/index.php?oldid=477411#Statistical_population (accessed on 10 March 2021).

4. The rate of risk from poverty *before* social transfers (with pensions *included* in the definition of social transfers) is calculated as the percentage of people (or thousands of people) who are at-risk-of-poverty, based on the equivalized disposable income before all social transfers—including pensions, over the total population.

Eurostat, https://ec.europa.eu/eurostat/statisticsexplained/index.php?oldid=477411#Statistical_population (accessed on 10 March 2021).

5. PM_2.5_ emissions (in kilograms per capita) show population-weighted emissions of PM_2.5_. Fine particulates (PM_2.5_) are those whose diameters are less than 2.5 micrometers. Particulates can be carried deep into the lungs where they can cause inflammation and a worsening of the condition of people with heart and lung diseases. The smaller the particles, the deeper they travel into the lungs, with more potential for harm. Air emissions accounts record the flows of residual gaseous and particulate materials emitted by resident units and flowing into the atmosphere. Air emissions accounts record emissions arising from all resident units (=economic activities), regardless of where these emissions actually occur geographically. A unit is said to be a resident unit of a country when it has a center of economic interest in the economic territory of that country, that is, when it engages for an extended period (1 year or more) in economic activities in that territory.

Eurostat, https://ec.europa.eu/eurostat/cache/metadata/en/env_ac_ainah_r2_esms.htm (accessed on 12 March 2021).

6. Mean annual exposure to PM_2.5_ pollution (in µg/m^3^) reflects the estimated annual mean exposure level of an average resident to outdoor particulate matter, expressed as population-weighted PM_2.5_ levels. The underlying PM_2.5_ estimates are taken from the Global Burden of Disease (GBD) 2019 project. They are derived by integrating satellite observations, chemical transport models, and measurements from ground monitoring station networks.

Global Heath Data Exchange (GHDX), http://ghdx.healthdata.org/gbd-results-tool (accessed on 10 March 2021).

7. Five morbidity indicators: The incidence in persons older than 65 years of the following diseases: a. cardiovascular disease: any disease of the circulatory system, namely the heart (cardio) or blood vessels (vascular). Includes ACS, angina, stroke, and peripheral vascular disease. Also known as circulatory disease. b. ischemic heart disease: also heart attack and angina (chest pain). Also known as coronary heart disease. c. chronic obstructive pulmonary disease (COPD): chronic respiratory diseases (CRDs) are diseases of the airways and other structures of the lung. d. asthma: it is a disease characterized by recurrent attacks of breathlessness and wheezing, which vary in severity and frequency from person to person. This condition is due to inflammation of the air passages in the lungs and affects the sensitivity of the nerve endings in the airways so they become easily irritated. In an attack, the lining of the passages swell causing the airways to narrow and reducing the flow of air in and out of the lungs. e. tracheal, bronchial, and lung cancer (hereinafter designated as “tracheal cancer” as shorthand covering all three types of cancer): tracheal cancer is cancer that forms in tissue of the airway that leads from the larynx (voice box) to the bronchi (large airways that lead to the lungs). Tracheal is also called windpipe. Bronchus cancer is cancer that begins in the tissue that lines or covers the airways of the lungs, including small cell and non-small cell lung cancer. Lung cancer is cancer that forms in tissues of the lung, usually in the cells lining air passages. The two main types are small cell lung cancer and non-small cell lung cancer. These types are diagnosed based on how the cells look under a microscope.

GHDX. https://www.cancer.gov/publications/dictionaries (accessed on 10 March 2021).

8. Five mortality indicators: The rate of premature death among persons older than 65 years from each of the preceding five diseases: number of people with incidence of cardiovascular diseases, ischemic heart disease, chronic obstructive pulmonary disease (COPD), asthma, and tracheal, bronchus, and lung cancer; ages above 65, according to the GBD study.

GHDX, http://ghdx.healthdata.org/gbd-results-tool (accessed on 10 March 2021).

9. Real gross domestic product (GDP) per capita is calculated as the ratio of real GDP to the average population of a specific year. GDP measures the value of total final output of goods and services produced by an economy within a certain period of time. It includes goods and services that have markets (or which could have markets) and products which are produced by general government and non-profit institutions.

Eurostat, Methodology and explanatory notes, https://ec.europa.eu/taxation_customs/sites/taxation/files/taxation_trends_report_2018_methodology.pdf (accessed on 12 March 2021).

10. Health-related government expenditures per capita tracks all health spending in a given country over a defined period of time regardless of the entity or institution that financed and managed that spending. It generates consistent and comprehensive data on health spending in a country, which in turn can contribute to evidence-based policymaking.

World Bank, https://data.worldbank.org/indicator/SH.XPD.GHED.PC.CD (accessed on 12 March 2021).

11. Environmentally related taxes as a percentage of GDP are important instrument for governments to shape relative prices of goods and services. The characteristics of such taxes included in the database (e.g., revenue, tax base, tax rates, exemptions, etc.) are used to construct the environmentally related tax revenues with a breakdown by environmental domain: energy products (including vehicle fuels); motor vehicles and transport services; measured or estimated emissions to air and water, ozone depleting substances, certain non-point sources of water pollution, waste management and noise, as well as management of water, land, soil, forests, biodiversity, wildlife, and fish stocks.

OECD, https://data.oecd.org/envpolicy/environmental-tax.htm (accessed on 1 March 2021).

12. Social security contributions as a percentage of GDP are paid on a compulsory or voluntary basis by employers, employees and self- and non-employed persons, shown as a percentage of GDP.

Eurostat, https://ec.europa.eu/eurostat/statisticsexplained/index.php?title=Glossary:Social_contributions (accessed on 22 February 2021).

13. Overall government spending per capita captures the burden imposed by government expenditures, which includes consumption by the state and all transfer payments related to various entitlement programs.

Index of Economic Freedom, https://www.heritage.org/index/about (accessed on 15 February 2021).

14. The corruption perception index scores and ranks countries/territories based on how corrupt a country’s public sector is perceived to be by experts and business executives. It is a composite index, a combination of 13 surveys and assessments of corruption, collected by a variety of reputable institutions. Corruption generally comprises illegal activities, which are deliberately hidden and only come to light through scandals, investigations, or prosecutions.

Transparency International, https://www.transparency.org/en/cpi (accessed on 15 February 2021).

15. The Gini coefficient of economic inequality is defined as the relationship of cumulative shares of the population arrange according to the level of equivalized disposable income, to the cumulative share of the equivalized total disposable income received by them.

Eurostat, https://ec.europa.eu/eurostat/databrowser/view/tessi190/default/table?lang=en (accessed on 13 February 2021).

We collected two additional variables. As explained in Section 2.1.1, we ultimately confined the use of data on the welfare cost of PM_2.5_ *alone* to the instrumental variable, two-stage least squares (IV2SLS) model. We ultimately excluded data on the welfare cost of PM_2.5_ and PM_10_ pollution *combined* altogether. Nevertheless, we document these data sources in the interest of completeness.

16. The welfare cost of premature deaths per capita among elderly persons from PM_2.5_ and PM_10_ *combined* shows the welfare cost of premature deaths per capita among elderly persons from PM_2.5_ and PM_10_. Fine and coarse particulates (PM_10_) are those whose diameter is less than 10 micrometers, while fine particulates (PM_2.5_) are those whose diameters are less than 2.5 micrometers. Particulates can be carried deep into the lungs where they can cause inflammation and a worsening of the condition of people with heart and lung diseases.

Eurostat, https://ec.europa.eu/eurostat/cache/metadata/en/t2020_rn210_esmsip2.htm (accessed on 10 March 2021).

17. The welfare cost of premature deaths per capita among elderly persons from PM_2.5_ *alone* shows data on mortality from exposure to environmental risks are taken from GBD (2019), Global Burden of Disease Study 2019 Results. Welfare costs are calculated using a methodology adapted from OECD (2017b), The Rising Cost of Ambient Air Pollution thus far in the 21st Century: Results from the BRIICS and the OECD Countries.

OECD, https://stats.oecd.org/Index.aspx?DataSetCode=EXP_MORSC (accessed on 1 March 2021).

## Appendix B

Appendix B displays results from the imputation of missing values for PM_2.5_ exposure (*exposure*); chronic obstructive pulmonary disease (COPD) mortality (*copd_death*); and mortality from tracheal, bronchial, and lung cancer (*tracheal_death*).

Figure A1a,b illustrate the imputation of two values for *exposure* for Ireland and Germany through LOESS and a linear spline. Figure A1c,d illustrate the use of LOESS and a cubic spline to impute missing values for *copd_death* mortality from COPD and tracheal, bronchial, and lung cancer. Figure A2 summarizes the imputation process through histograms of all variables with imputed values, with “before” raw observations at left in blue and “after” imputed values at right in red.
